# Supplementary material for: A brain-derived insulin signal encodes protein satiety for nutrient-specific feeding inhibition
Source: Cell Rep. 2024 May 24;43(6):114282. doi: 10.1016/j.celrep.2024.114282 (PMC11220824; doi:10.1016/j.celrep.2024.114282)
Supplement: Document S1. Figures S1‒S7 [file mmc1.pdf]

Cell Reports, Volume *43*

Supplemental information

A brain-derived insulin signal encodes protein satiety for nutrient-specific feeding inhibition

Xiaoyu Li, Yang Yang, Xiaobing Bai, Xiaotong Wang, Houqi Tan, Yanbo Chen, Yan Zhu, Qili Liu, Mark N. Wu, and Yan Li

SUPPLEMENTAL INFORMATION

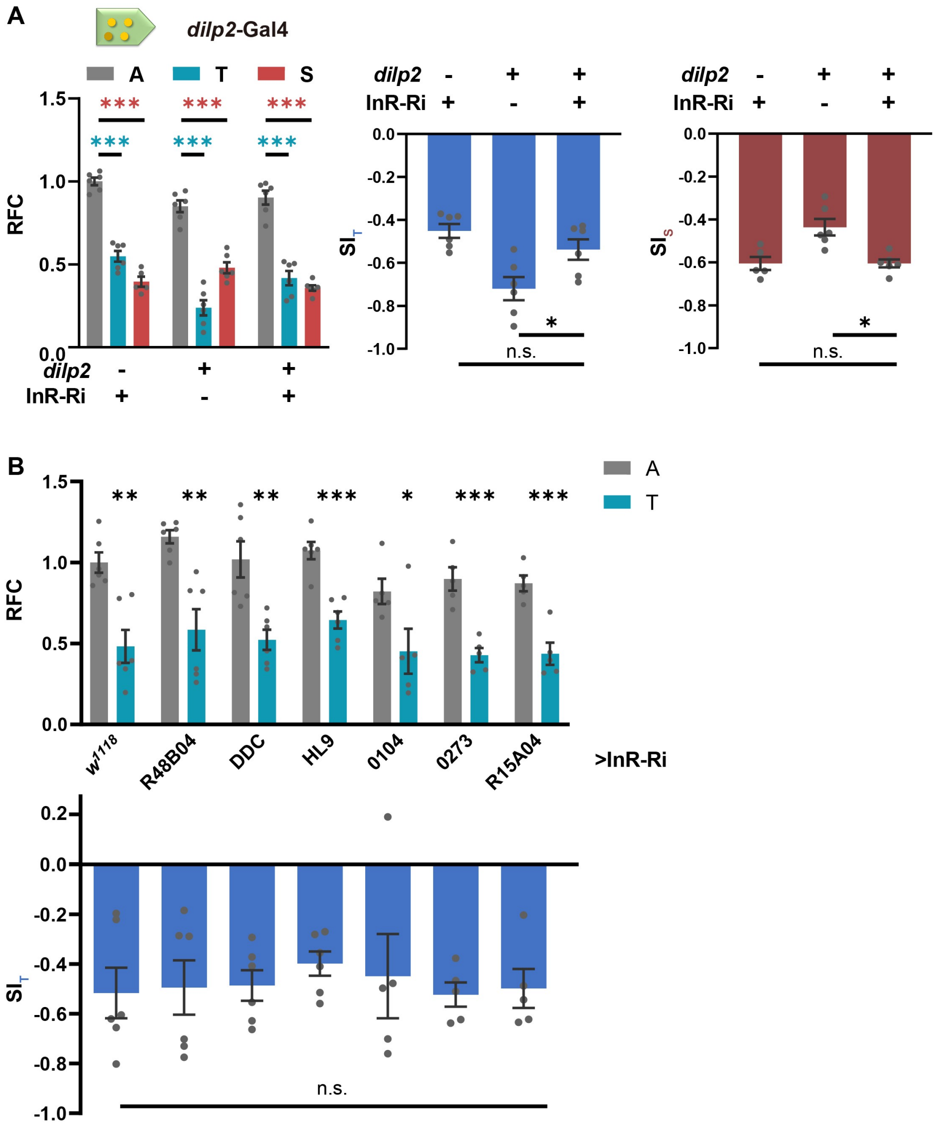

Figure S1. Insulin signalling in IPCs and some DANs is not required for feeding inhibition.

Related to Figure 1.

(A) Knockdown of InR in IPCs did not affect the PIFI or SIFI effect. n = 5-6.

(B) Knockdown of InR in various subsets of DANs did not affect the PIFI effect. n = 5-6.

n represents the number of trials. Student's *t* test for Relative Food Consumption (RFC). One-way ANOVA, Dunnett test for Suppression Index (SI). *, $p < 0.05$. **, $p < 0.01$. ***, $p < 0.001$. n.s. indicates no statistical significance. The data are shown in Mean ± S.E.M.

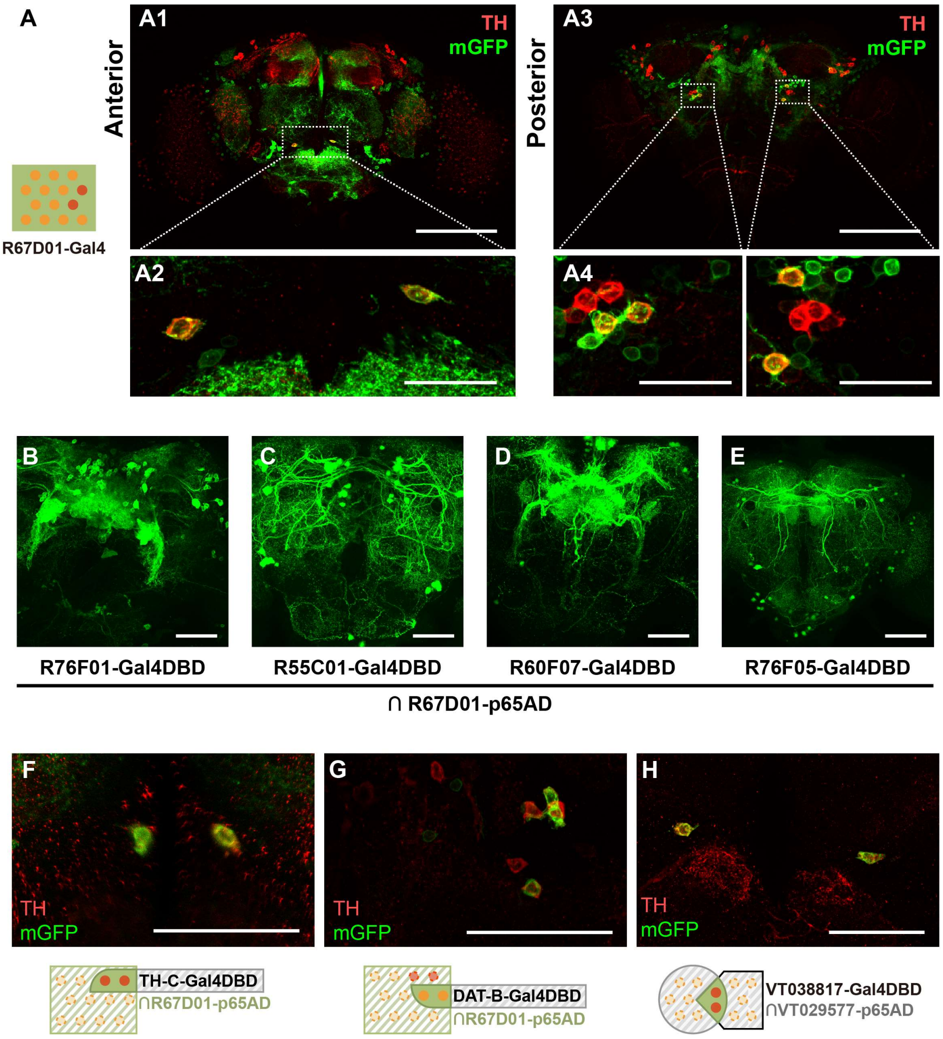

Figure S2. Identification of two types of DANs in R67D01-labeled neurons. Related to Figure 2.

(A) TH staining (red) marks the cell bodies of two types of DANs in R67D01-neurons from the anterior view (A1) and the posterior view (A3). Scale bar, 100 μm. A2 and A4 are the partial enlarged view of A1 and A3. Scale bar, 20 μm.

(B-E) The expression pattern of the split-Gal4 lines of R67D01-p65AD in combination with different Gal4DBD fly strains. Scale bar, 50 μm.

(F-H) The enlarged view of cell bodies of T1-Gal4 (Fig. 2A), PPM3-Gal4 (Fig. 2B) and T1'-Gal4 (Fig. 2C). Scale bar, 50 μm.

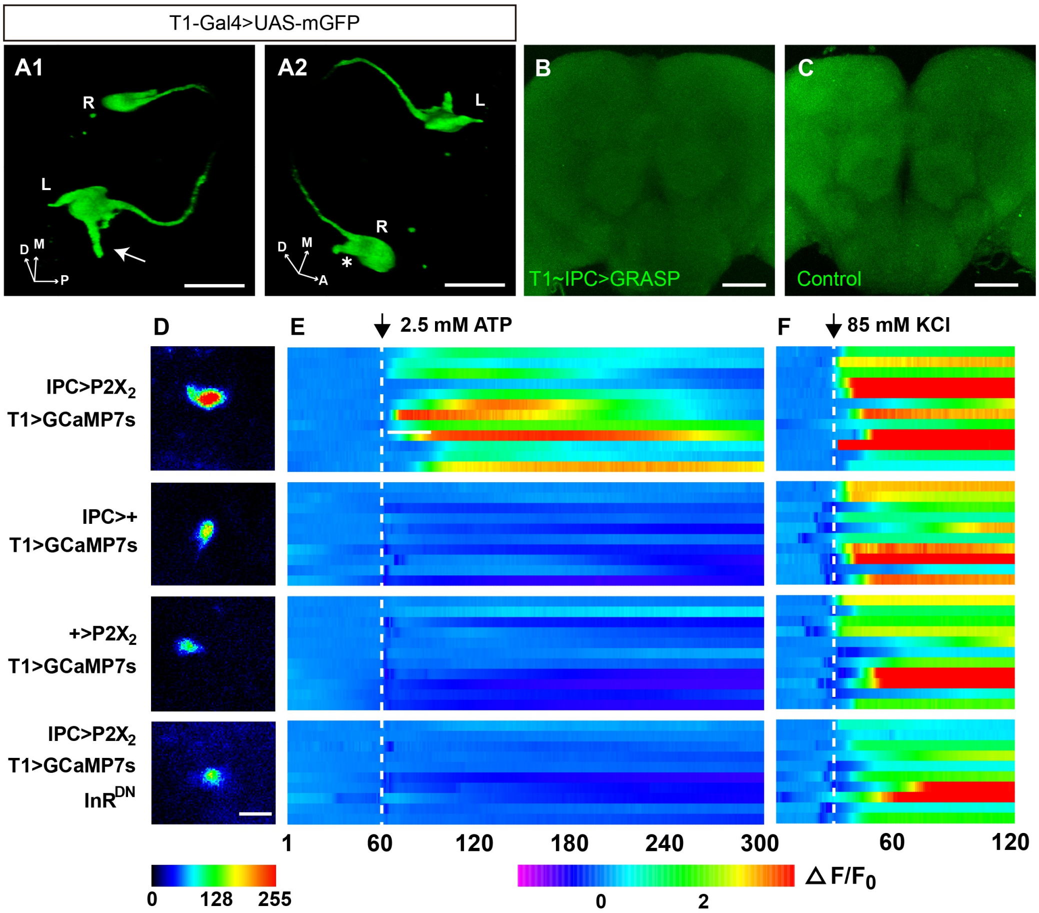

Figure S3. No synaptic but functional connection between IPCs and T1-DANs. Related to Figure 3.

(A) The 3D illustration of filipodia-like (arrow) and lamellipodia-like (star) cellular protrusion of T1-DANs. R and L indicates the right and left side, respectively. Scale bar, 10 µm.

(B-C) There was no detectable GRASP signal between IPCs and T1-DANs. Scale bar, 50 µm.

(D) Activation of IPCs induced a remarkable increase in calcium levels of T1-DANs. Representative images in different groups. Scale bar, 15 µm.

(E-F) Each row in the heat map represents the $\Delta F/F_0$ response of one T1-DANs neuron. Each pair of vertical dashed lines marks stimulation duration. The number of brains n = 10-12.

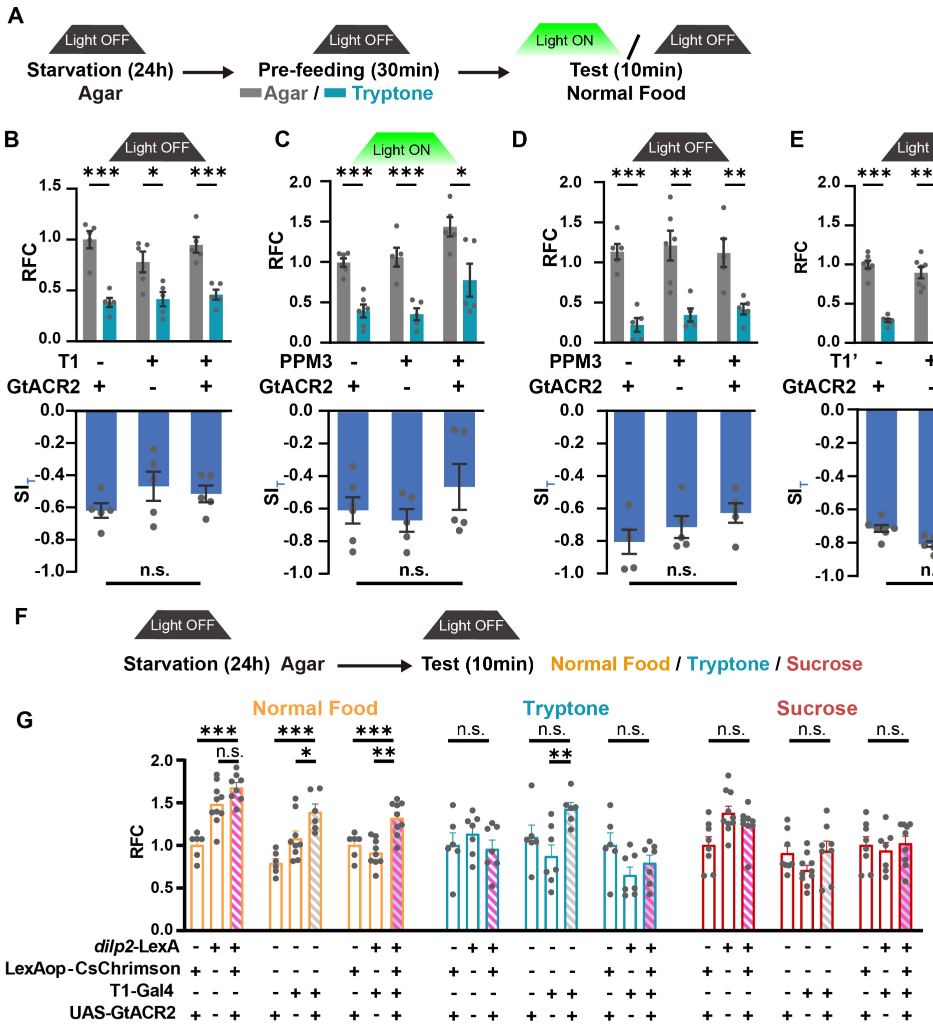

Figure S4. Silencing PPM3-DANs does not affect the PIFI effect. Related to Figure 4.

(A) The diagram of optogenetic manipulation in the pre-feeding paradigm.

(B) Flies showed comparable PIFI when light was OFF. n = 5.

(C-D) Inhibition of PPM3-DANs does not affect PIFI. n = 5-6.

(E) Flies showed comparable PIFI when light was OFF. n = 6-7.

(F-G) Flies showed comparable food consumption when light was OFF. n = 6-10. Same set of data were used for the first and seventh column in all the three groups of experiments.

n represents the number of trials. Student's *t* test for Relative Food Consumption (RFC) in B-E. One-way ANOVA, Dunnett test for Suppression Index (SI) in B-E and RFC in G. See also Table S2 for Two-way ANOVA comparison between groups. *, $p < 0.05$. **, $p < 0.01$. ***, $p < 0.001$. n.s. indicates no statistical significance. The data are shown in Mean ± S.E.M.

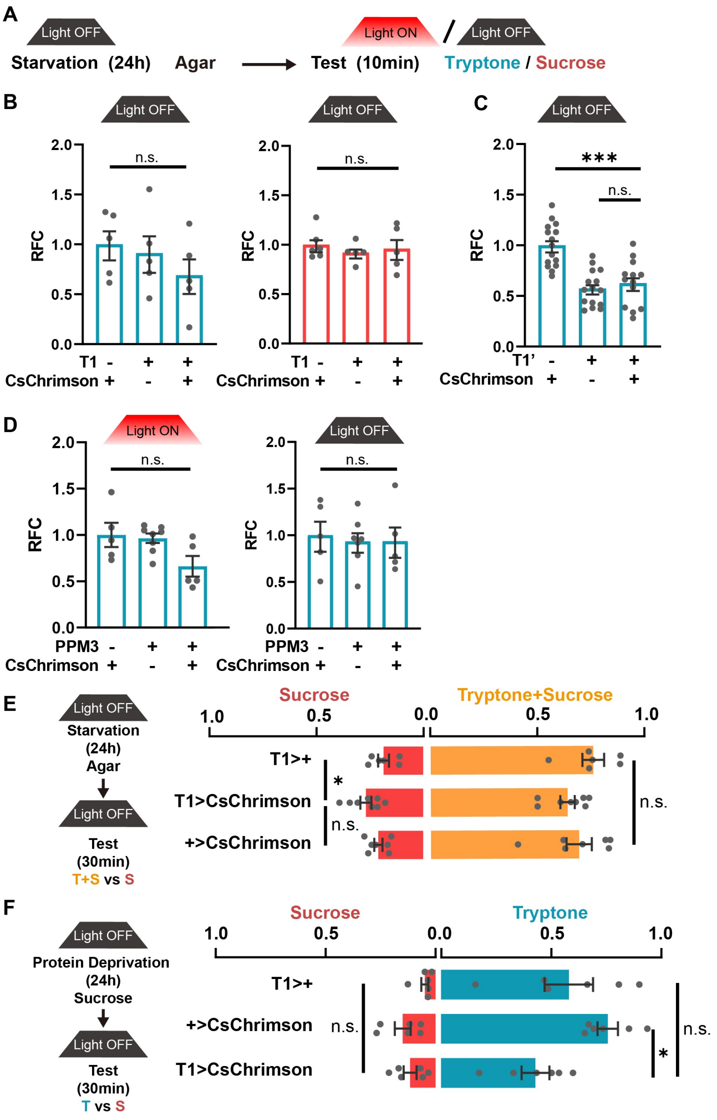

Figure S5. Activation of PPM3-DANs does not affect protein feeding. Related to Figure 5.

(A) The diagram of optogenetic manipulation in the feeding test.

(B-C) Flies showed comparable food consumption when light was OFF. n = 5-6 in B. n = 13-15 in C.

(D) Opto-activating PPM3-DANs did not affect protein intake. n = 5-8.

(E-F) Flies showed comparable choice index when light was OFF. n = 6-8.

n represents the number of trials. One-way ANOVA, Dunnett test for Relative Food Consumption (RFC) in B-D and Choice Ratio in E and F. *, $p < 0.05$. **, $p < 0.01$. ***, $p < 0.001$. n.s. indicates no statistical significance. The data are shown in Mean ± S.E.M.

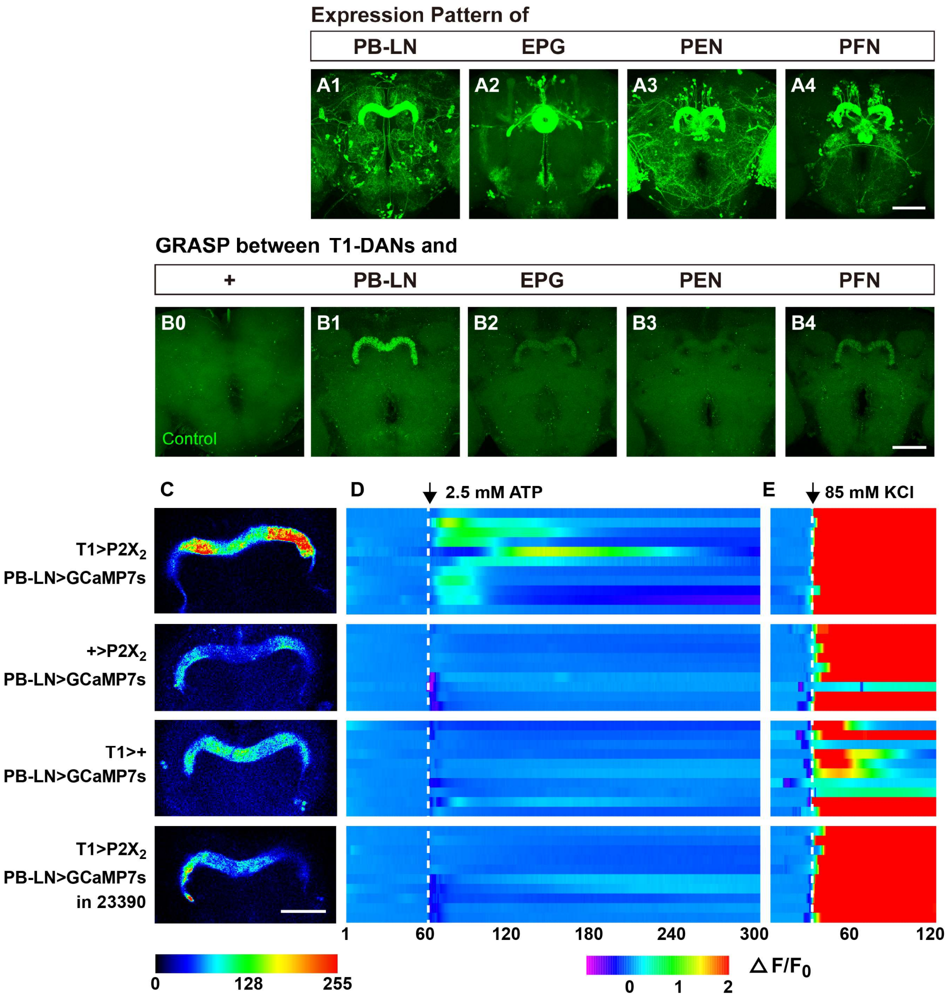

Figure S6. T1-DANs form synaptic and functional connections with PB-LNs. Related to Figure 6.

(A) The expression pattern of different types of PB-projecting neurons.

(B) The syb:GRASP between T1-DANs and PB-projecting neurons (B1-B4). B0 is the control with no PB-driver.

(C) Activation of T1-DANs induced a remarkable increase in calcium levels of PB-LNs. Representative images in different groups.

(D-E) Each row in the heat map represents the $\Delta F/F_0$ response of individual PB-LNs. Each pair of vertical dashed lines marks stimulation duration. The number of brains n = 9-11.

Scale bar, 50 μm.

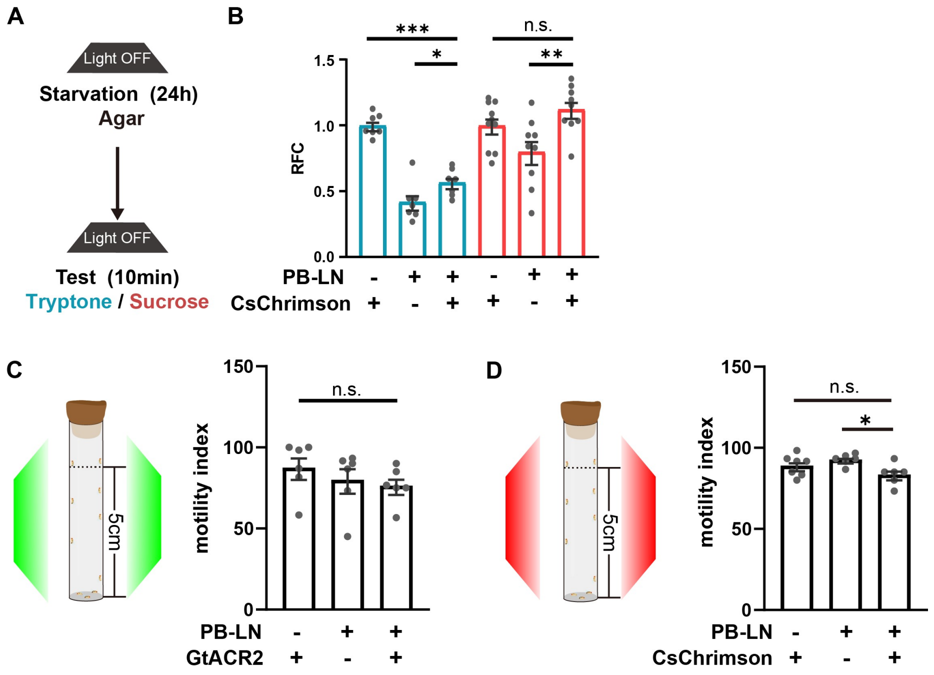

Figure S7. Manipulation of PB-LNs does not affect motility. Related to Figure 7.

(A-B) Flies showed comparable food consumption when light was OFF. n = 7-10.

(C) Silencing PB-LNs did not affect the climbing activity of flies. n = 6.

(D) Optogenetic activation of PB-LNs did not affect the climbing activity of flies. n = 6-7.

n represents the number of trials. One-way ANOVA, Dunnett test. *, $p < 0.05$. **, $p < 0.01$. ***, $p < 0.001$. n.s. indicates no statistical significance. The data are shown in Mean ± S.E.M.
