# Supplementary material for: A brain-derived insulin signal encodes protein satiety for nutrient-specific feeding inhibition
Source: Cell Rep. 2024 May 24;43(6):114282. doi: 10.1016/j.celrep.2024.114282 (PMC11220824; doi:10.1016/j.celrep.2024.114282)
Supplement: Document S2. Article plus supplemental information [file mmc7.pdf]

Cell Reports

Article

A brain-derived insulin signal encodes protein satiety for nutrient-specific feeding inhibition

Graphical abstract

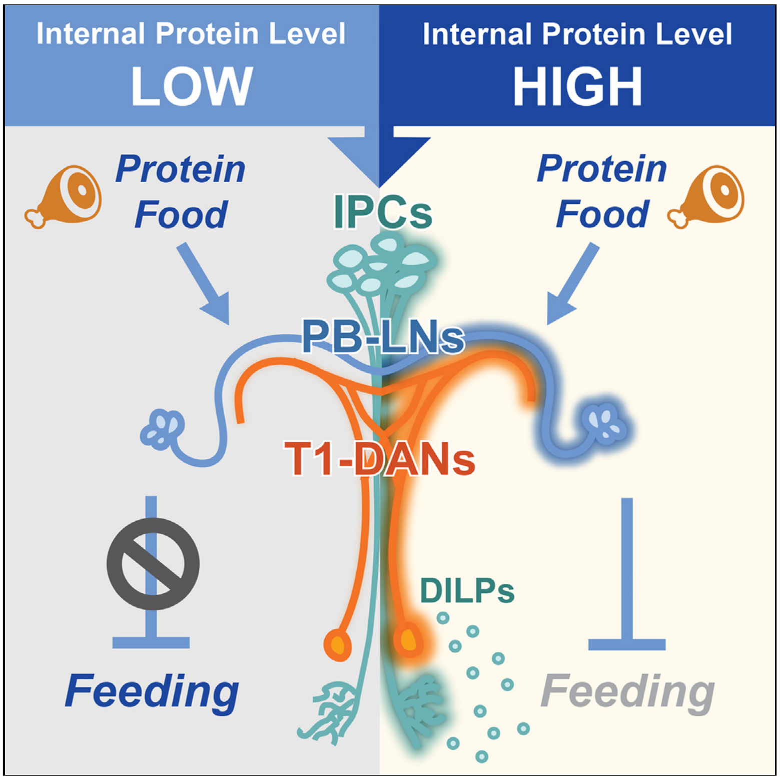

Highlights

- Insulin signaling in a pair of T1-DANs represents protein-specific satiety
- In female flies, protein intake induces DILP2 release and activates T1-DANs
- Opto-activating IPCs in the brain suppresses food intake of both sugar and protein
- Downstream of IPCs, the T1-PB circuit specifically mediates protein satiety signal

Authors

Xiaoyu Li, Yang Yang, Xiaobing Bai, ..., Qili Liu, Mark N. Wu, Yan Li

Correspondence

liyan@ibp.ac.cn

In brief

In this study, Li et al. identify a neural circuit in adult female fly brain that functions downstream of insulin-producing cells and selectively responds to the protein-intake-induced insulin signal. Through this circuit, such an insulin-encoded protein satiety signal suppresses feeding behavior, specific to protein intake.

Li et al., 2024, Cell Reports *43*, 114282
June 25, 2024 © 2024 The Authors. Published by Elsevier Inc.
https://doi.org/10.1016/j.celrep.2024.114282

CellPress

Cell Reports

CellPress
OPEN ACCESS

Article

A brain-derived insulin signal encodes protein satiety for nutrient-specific feeding inhibition

Xiaoyu Li,[1,2,6] Yang Yang,[1,2,6] Xiaobing Bai,[1,2,3,6] Xiaotong Wang,[1,2] Houqi Tan,[1,2] Yanbo Chen,[1] Yan Zhu,[1,2,3] Qili Liu,[4] Mark N. Wu,[5] and Yan Li[1,2,3,7,*]
[1]Institute of Biophysics, State Key Laboratory of Brain and Cognitive Science, Center for Excellence in Biomacromolecules, Chinese Academy of Sciences, Beijing 100101, China
[2]University of Chinese Academy of Sciences, Beijing 100049, China
[3]Sino-Danish Center for Education and Research, Beijing 100190, China
[4]Department of Anatomy, University of California, San Francisco, San Francisco, CA 94158, USA
[5]Department of Neurology, Johns Hopkins University School of Medicine, Baltimore, MD 21205, USA
[6]These authors contribute equally
[7]Lead contact
*Correspondence: liyan@ibp.ac.cn
https://doi.org/10.1016/j.celrep.2024.114282

SUMMARY

The suppressive effect of insulin on food intake has been documented for decades. However, whether insulin signals can encode a certain type of nutrients to regulate nutrient-specific feeding behavior remains elusive. Here, we show that in female *Drosophila*, a pair of dopaminergic neurons, tritocerebrum 1-dopaminergic neurons (T1-DANs), are directly activated by a protein-intake-induced insulin signal from insulin-producing cells (IPCs). Intriguingly, opto-activating IPCs elicits feeding inhibition for both protein and sugar, while silencing T1-DANs blocks this inhibition only for protein food. Elevating insulin signaling in T1-DANs or opto-activating these neurons is sufficient to mimic protein satiety. Furthermore, this signal is conveyed to local neurons of the protocerebral bridge (PB-LNs) and specifically suppresses protein intake. Therefore, our findings reveal that a brain-derived insulin signal encodes protein satiety and suppresses feeding behavior in a nutrient-specific manner, shedding light on the functional specificity of brain insulin signals in regulating behaviors.

INTRODUCTION

Insulin is the most important postprandial signal for regulating metabolic and physiological processes.[1,2] Interestingly, after the consumption of different types of macronutrients, insulin levels are found to change in different manners. The circulating insulin is greatly increased after the consumption of carbohydrates and protein[3] but not lipid,[4] while insulin levels in the hypothalamus are increased upon carbohydrates intake, decreased upon lipid intake, and unchanged upon protein intake.[3,4] These observations suggest that insulin signals discriminatively respond to different types of macronutrients. In addition to receiving peripheral insulin,[5,6] the brain has also been found to produce and release insulin, as well as insulin-like growth factors (IGFs).[7–10] Notably, both the insulin receptor (InR) and IGF receptors (IGFRs) are widely distributed in the brain and can form hybrid receptors.[11,12] Moreover, both insulin and IGF1 can bind to the InR and IGFRs with different affinities.[13–16] Such complex organization of the ligand sources, receptors, and crosstalk raises the speculation that instead of a general nutrition state, different insulin signals in the brain may represent different types and/or levels of nutrients for precise metabolic and behavioral regulation.

In the central nervous system (CNS), accumulating evidence shows that the insulin signal participates in the regulation of various innate[17,18] and cognitive behaviors.[19,20] Among these regulations, the most prominent function of insulin is reducing food intake. Since the 1970s, intraventricular or intranasal administration of insulin has been found to significantly decrease food intake in baboons, marmots, and rats.[21–24] Notably, such feeding suppression appears independent of the metabolic regulation function of insulin.[21] In agreement with these insulin administration studies, whole-brain knockout (KO) of the InR results in a significant increase in the food intake of mice.[25] Furthermore, conditional KO of the InR in the orectic Agouti-related protein neurons also promotes feeding.[26] Nevertheless, a recent study reported that activating insulin-expressing neurons in the hindbrain promotes feeding.[27] In *Drosophila*, most studies are in line with the notion that insulin signals are anorexigenic,[28–33] whereas an orectic effect of insulin signaling has also been reported.[34–36] In the brain, insulin-producing cells (IPCs) produce three types of *Drosophila* insulin-like

This is an open access article under the CC BY license (http://creativecommons.org/licenses/by/4.0/).

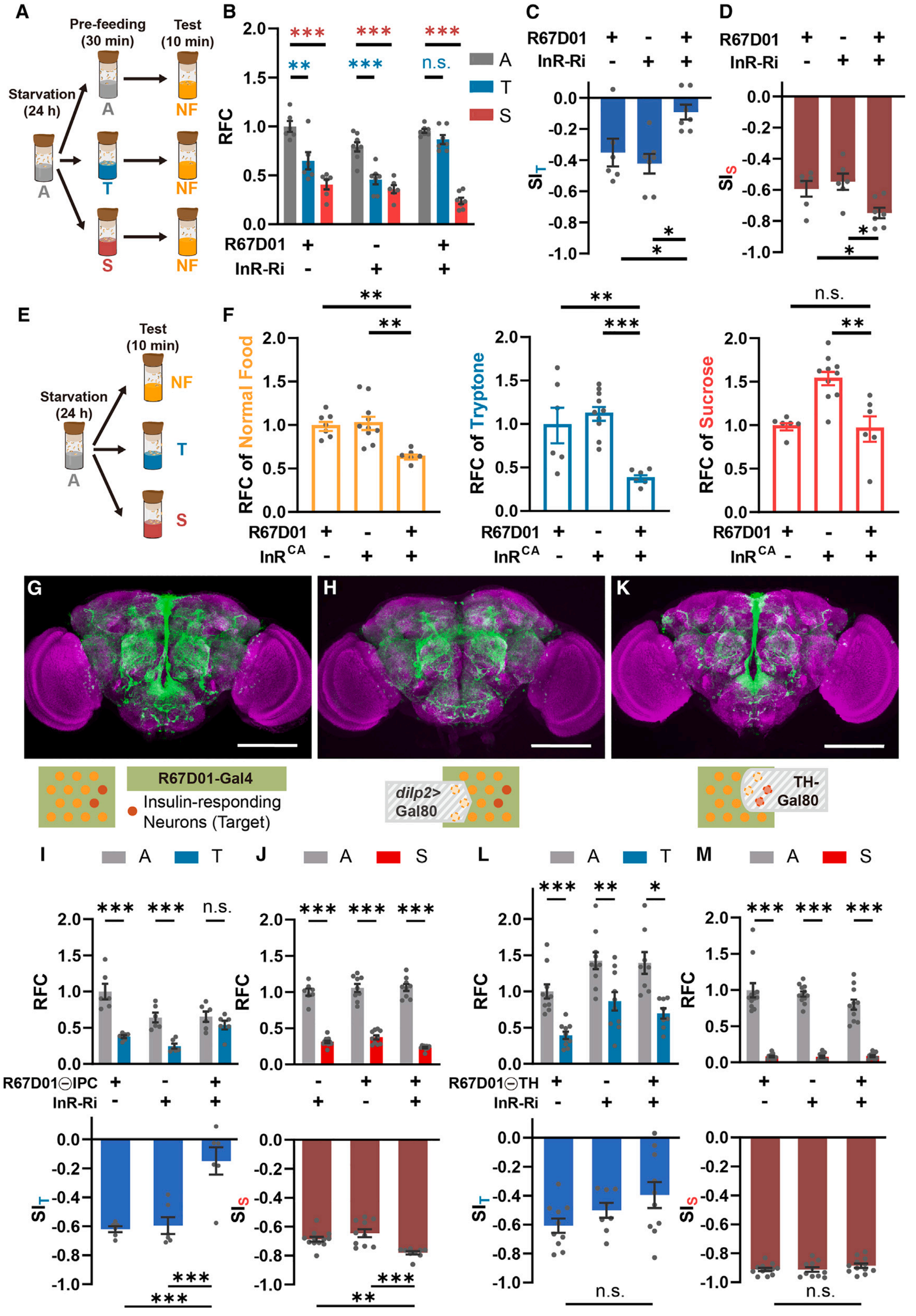

(legend on next page)

peptides (DILPs), and intriguingly, the expression levels of them vary with the duration of starvation in different ways.[37] These findings suggest that insulin signals possess complex function in regulating feeding behavior, which relies on the specific neural circuits.

Out of the three macronutrients, protein elicits the strongest satiety. Notably, protein consumption triggers a remarkable increase in circulating insulin to a comparable level to that post-sugar consumption.[38,39] Our earlier study implied that brain insulin signaling is required for protein-intake induced feeding inhibition (PIFI) in adult female flies.[40] To determine whether there is an insulin signal representing protein-specific satiety, we screened for potential downstream neurons in which insulin signaling is responsible for PIFI. Remarkably, we found that a single pair of dopaminergic neurons, tritocerebrum 1-dopaminergic neurons (T1-DANs), are necessary and sufficient to respond to the insulin signal triggered by protein overeating, thereby achieving protein-specific feeding inhibition within the time window of a meal. Moreover, we delineated the downstream circuit of T1-DANs in the protocerebral bridge (PB), where this protein satiety signal is integrated with protein food information for eliciting feeding suppression.

RESULTS

Insulin signaling in R67D01-labeled DANs is required for PIFI

To study insulin signals in the regulation of PIFI, we knocked down the InR using different Gal4 lines and subjected these flies to the pre-feeding paradigm (Figure 1A). As shown in Figure 1B, pre-feeding of protein food (tryptone) suppressed the following consumption of mixed food (normal food) in control flies; however, this suppression was abolished when the InR was knocked down in R67D01-Gal4-labeled neurons (R67D01-Ns). The statistical results showed that the suppression index of protein food (SI_T) was significantly decreased in these InR-knockdown (KD) flies compared with the two control groups (Figure 1C). In contrast, pre-feeding of sugar food (sucrose) elicited a comparable suppression effect, termed sugar-intake induced feeding inhibition (SIFI), in InR-KD and control groups (Figure 1B), and the suppression index of sugar food (SI_S) was not decreased in these InR-KD flies (Figure 1D).

We further tested whether the insulin signal in R67D01-Ns is sufficient to suppress food intake. As shown in Figures 1E and 1F, the expression of the constitutively active form of InR (InR^{CA})[41] in R67D01-Ns resulted in a significant feeding suppression of normal food in fasted flies. Moreover, these flies exhibited a significant reduction in tryptone, but not sucrose, consumption (Figure 1F). Together, these results indicate that R67D01-Ns include the neurons that are responsible for the insulin-mediated PIFI effect.

We then examined the expression pattern of R67D01-Gal4 by expressing membrane-GFP (mGFP). As shown in Figure 1G, R67D01-Gal4 labels a number of neurons, among which IPCs are the most apparent. The InR has been found to be expressed in IPCs, and this insulin signaling feedback regulates the expression of DILPs.[42] However, knocking down the InR in IPCs affected neither PIFI nor SIFI (Figure S1A). In addition, we suppressed the expression of InR-RNAi in IPCs using *dilp2*-LexA>LexAop-Gal80 (Figure 1H); thus, the InR was knocked down in the remaining R67D01-Ns. These flies displayed impaired PIFI but normal SIFI, shown as a significant decrease in the SI_T but not the SI_S (Figures 1I and 1J), which is similar to the flies with InR KD in all R67D01-Ns (Figures 1A–1C). We further utilized TH-Gal80 to block the Gal4 function in DANs (Figure 1K). Notably, InR KD in the remaining R67D01-Ns showed no effect on either PIFI or SIFI (Figures 1L–1M), indicating that the R67D01-labeled DANs are required for PIFI.

T1-DANs are responsible for insulin-signal-mediated feeding suppression

To find out which group of DANs is responsible for mediating the insulin signal to regulate PIFI, we examined several Gal4 fly strains that label different groups of DANs. However, InR KD in these DANs did not block the PIFI effect (Figure S1B). We then searched for R67D01-DANs by performing immunostaining against tyrosine hydroxylase (TH), and two groups of R67D01 neurons were found to be TH positive. As shown in Figure S2A, one pair of DANs locate close to the esophageal foramen showing in the anterior view, and the other group of DANs locate at the posterior dorsal region.

To obtain a restricted expression pattern of these DANs, we utilized the split-Gal4 approach.[43] We generated the R67D01-p65AD transgenic fly and combined it with various Gal4DBD fly strains labeling different subsets of DANs. Among these split-Gal4 strains, we found that two of them, R67D01-p65AD;

Figure 1. Insulin signaling in R67D01-labeled DANs is required for PIFI

(A) The diagram of pre-feeding paradigm. During pre-feeding, tryptone (T) was used as the protein food, sucrose (S) as the sugar food, and agar (A) as the no-pre-feeding control. Normal food (NF) was used in the test.

(B–D) Knocking down InR in R67D01 neurons abolished the feeding inhibition effect induced by protein pre-feeding (PIFI) but not sugar pre-feeding (SIFI). The suppression index of protein pre-feeding (SI_T) in these InR-KD flies was significantly decreased (C), whereas that of sugar pre-feeding (SI_S) was not impaired (D). n = 6–8.

(E and F) In hungry flies, expressing InR^{CA} in R67D01 neurons reduced food consumption of normal food and tryptone but not sucrose. n = 6–10.

(G) The expression pattern of R67D01-Gal4. Dots symbolize the R67D01-labeled neurons, and the red dot represents the insulin-responding neurons that are required for PIFI.

(H–J) InR KD in R67D01ΘIPC neurons (with no expression in IPCs, H) abolished the PIFI effect (I). n = 6–12.

(K–-M) InR KD in R67D01ΘTH neurons (with no expression in DANs, K) did not affect the PIFI (L) or SIFI (M) effect. n = 8–12.

n represents the number of trials. Student's t test for relative food consumption (RFC) in (B), (I), (J), (L), and (M). One-way ANOVA, Dunnett test for suppression index (SI) in (C), (D), (I), (J), (L), and (M) and for RFC in (F). $^*p < 0.05$, $^{**}p < 0.01$, and $^{***}p < 0.001$. n.s. indicates no statistical significance. The data are shown in mean ± SEM. Scale bar, 100 μm.

See also Figure S1.

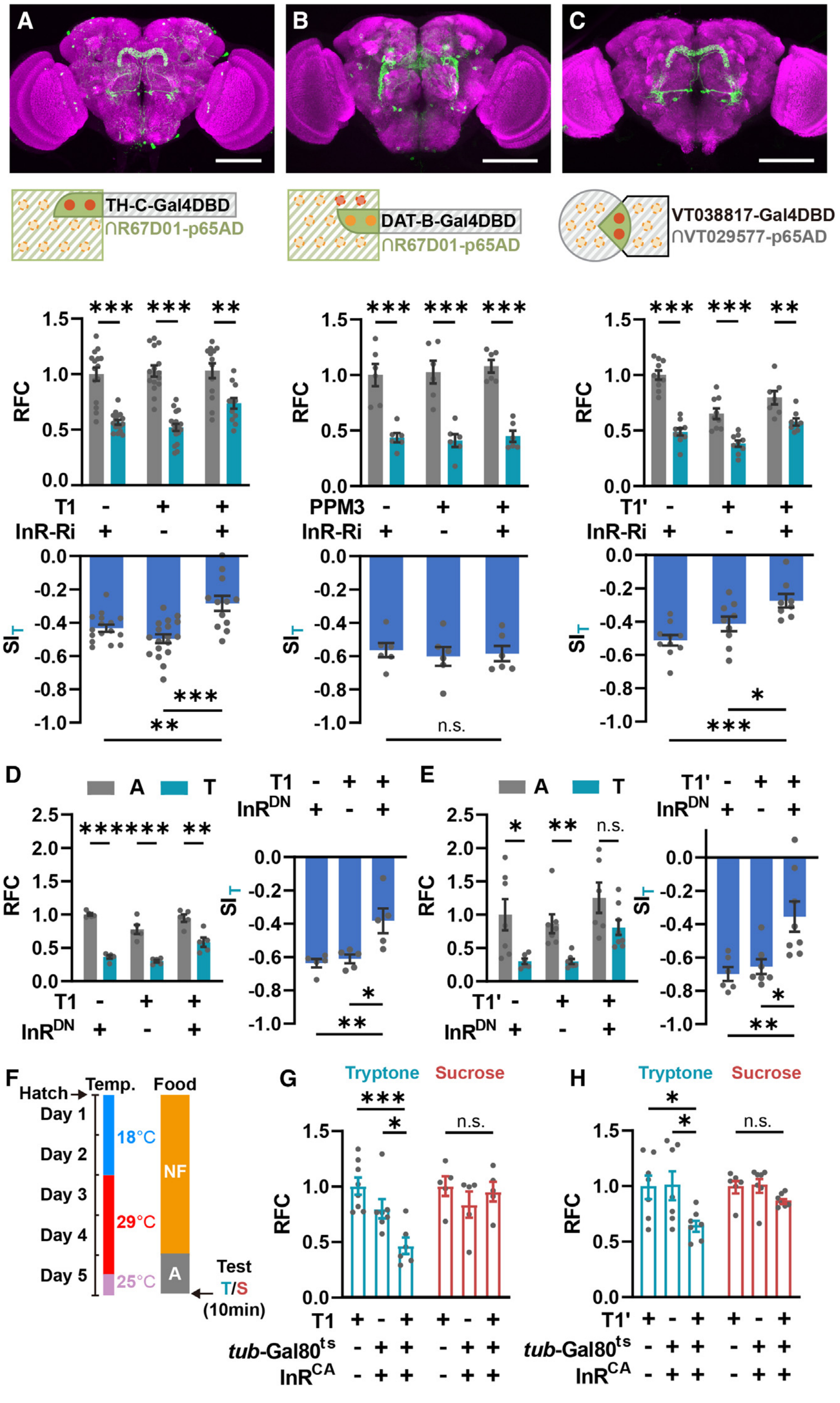

Figure 2. Insulin signaling in T1-DANs is required for the PIFI effect

(A) The PIFI effect was significantly reduced in flies with InR KD in T1-DANs using T1-Gal4. $n = 12–18$.

(B) InR KD in PPM3-DANs did not affect PIFI effect. $n = 6$.

(C) InR KD in T1-DANs by T1′-Gal4 suppressed the PIFI effect. $n = 7–10$.

(D and E) The PIFI effect was significantly decreased when expressing InR^{DN} in T1-DANs using T1- or T1′- Gal4 strains. $n = 5$ in (D). $n = 6–8$ in (E).

(F) The flow diagram of temperature-induced gene expression. Flies were collected at hatch, cultivated in NF at 18°C for 2 days, and then transferred to 29°C to induce gene expression. After another 2 days, flies were transferred to agar for 24 h starvation (12 h at 29°C and 12 h at 25°C) and were subjected to the feeding test at 25°C.

(G and H) Expressing InR^{CA} in T1-DANs during adult stage reduced food consumption of tryptone but not sucrose. $n = 5–8$.

n represents the number of trials. Student's t test for RFC in (A)–(E). One-way ANOVA, Dunnett test for SI in (A)–(E) and for RFC in (G) and (H). $^{*}p < 0.05$, $^{**}p < 0.01$, and $^{***}p < 0.001$. n.s. indicates no statistical significance. The data are shown in mean ± SEM. Scale bar, 100 μm.

See also Figure S2.

TH-C-Gal4DBD and R67D01-p65AD;DAT-B-Gal4DBD, showed apparent expression in the two types of R67D01-DANs, respectively (Figures 2A and 2B, top, and S2B–S2G). According to the position of cell bodies and the morphology of neural projection, we recognized them as T1 and PPM3 (protocerebral posterior median 3) DANs and named them as T1-Gal4 and PPM3-Gal4, respectively.

In the PIFI assay, knocking down the InR in T1-DANs resulted in a significant decrease in the SI_T, whereas InR KD in PPM3-DANs had no effect on the SI_T (Figures 2A and 2B, bottom). We further obtained an independent split-Gal4 fly strain, VT029577-p65AD;VT038817-Gal4DBD, which specifically labels T1-DANs, named T1′-Gal4 (Figures 2C and S2H). Consistently, knocking down the InR using T1′-Gal4 also significantly suppressed the PIFI effect (Figure 2C).

We then utilized the dominant-negative form of InR (InR^{DN})[41] as an alternative approach to block insulin signaling. Consistent with the results obtained from InR-RNAi flies, expressing InR^{DN} in T1-DANs led to a significant decrease in the SI_T (Figures 2D and 2E). In contrast, increasing insulin signaling in T1-DANs by expressing InR^{CA} suppressed protein intake in hungry flies (Figures 2F–2H). Together, these results indicate that insulin signaling in the T1-DANs is required for the PIFI and that elevating insulin signaling in the T1-DANs is sufficient to suppress feeding selectively for protein food.

T1-DANs receive insulin signal directly from IPCs

We then examined whether T1-DANs were activated upon protein consumption using the CaMPARI approach.[44] As shown in Figures 3A–3C and Table S1, the calcium levels (indicated by

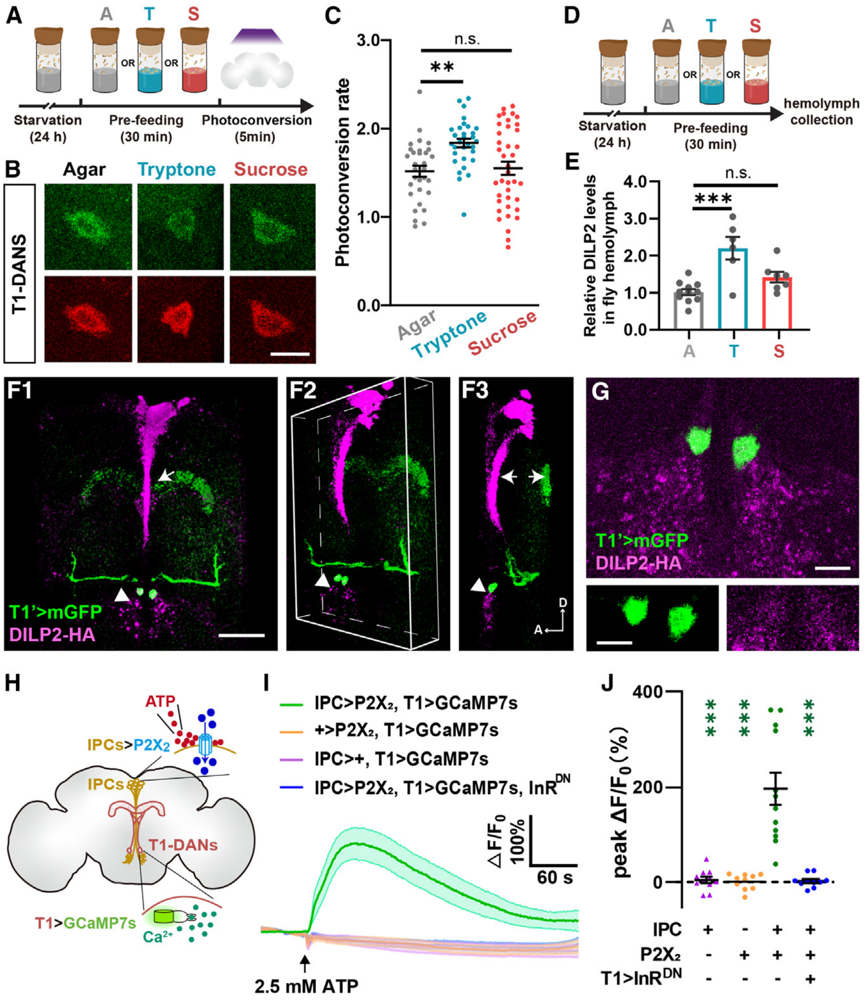

Figure 3. T1-DANs are activated by IPC-derived insulin signals after protein consumption

(A)The diagram of feeding treatment and photoconversion for the CaMPARI experiment.

(B and C) Protein feeding induced a significant increase in the neural activity of T1-DANs. The number of brains: n = 31–39. Scale bar, 10 μm in (B). See also Table S1.

(D and E) Protein, but not sugar, consumption triggered the secretion of DILP2. The number of trials: n = 6–11.

(F and G) T1-DANs are close to IPCs at the axon bundle (arrow) and projection terminal (arrowhead) regions from the anterior view (D1). When the 3D image turns 45° (D2) or 90° (D3), only the cell bodies of T1-DANs are close the IPC projection region (arrowhead), which is enlarged in (E). Scale bars, 50 μm in (F) and 10 μm in (G).

(H–J) Pharmacological activation of IPCs induced a remarkable increase in calcium levels of T1-DANs, and this increase was abolished when insulin signaling was blocked in T1-DANs. The cell body region of one T1-DAN in each brain was selected as the region of interest (ROI). The number of brains: n = 10–12.

One-way ANOVA, Dunnett test in (C), (E), and (J). $^*p < 0.05$, $^{**}p < 0.01$, and $^{***}p < 0.001$. n.s. indicates no statistical significance. The data are shown in mean ± SEM.

See also Figure S3 and Video S1.

the photoconversion rate) were significantly increased after 30-min protein consumption, in comparison with that in the agar group. In contrast, sugar consumption for 30 min did not affect the calcium levels in the T1-DANs. Thus, T1-DANs are selectively activated by protein feeding. To determine whether protein intake triggers the release of DILPs, we utilized the ELISA method.[45] As shown in Figures 3D and 3E, the DILP2 levels in the hemolymph were significantly elevated after protein consumption, but not after sugar consumption, in adult female flies.

In the *Drosophila* brain, DILP2 is generated by a group of endocrine neurons, namely IPCs. To determine the spatial relationship between T1-DANs and IPCs, we labeled T1-DANs by expressing mGFP and IPCs by DILP2-HA immunostaining.[45] From the front view, the two neurons overlapped in the cell body and projection areas of T1-DANs, respectively (Figure 3F1). By observing the 3D reconstructed image from two angles, 45° and 90° (Figures 3F2 and 3F3), we found that T1 projection is distant from the axon bundle of IPCs, while the cell bodies of T1-DANs are adjacent to the IPC projection terminals (Figure 3G). Interestingly, we observed cellular protrusions on the cell bodies of T1-DANs, appearing as filipodia-like and lamellipodium-like structures (Figure S3A; Video S1). Nevertheless, we did not detect any reconstituted GRASP signal[46] between IPCs and T1-DANs (Figures S3B and S3C). These results suggested that T1-DANs receive insulin signal through short-range paracrine rather than synaptic transmission.

Next, we asked whether T1-DANs are directly activated by IPCs. As shown in Figure 3H, $P2X_2$ was expressed in IPCs to allow the pharmacological activation of these neurons by puffing ATP,[47,48] and GCaMP7s was expressed in T1-DANs for calcium imaging. Tetrodotoxin (TTX) was added to the bath to block the potential indirect activation of T1-DANs through other neurons.[49] The results showed that activating IPCs led to a fast and significant increase of the calcium signals in T1-DANs, while genetic control groups lacking either the Gal4 or the $P2X_2$ displayed no response (Figures 3I, 3J, and S3D–S3F). Moreover, this activation was abolished when the InR was knocked down in T1-DANs, indicating that T1-DANs receive the insulin signal directly from IPCs.

Activation of T1-DANs is necessary and sufficient for PIFI

To determine whether the activation of T1-DANs is required for PIFI, we utilized the optogenetic approach by expressing GtACR2 in T1-DANs.[50] The results showed that silencing this single pair of T1-DANs completely blocked the PIFI effect,

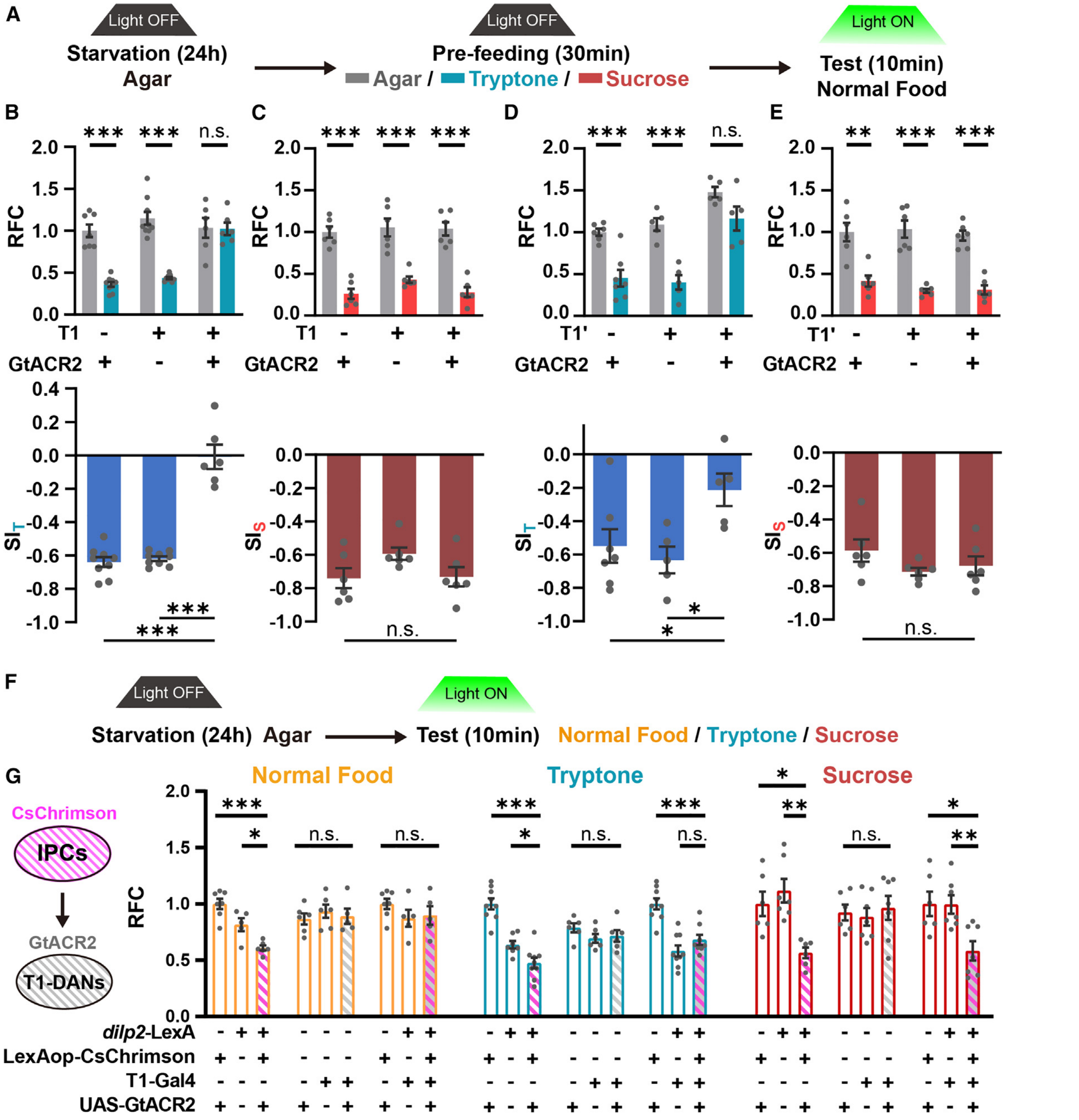

Figure 4. PIFI is abolished when T1-DANs are inhibited

(A) The diagram of optogenetic manipulation in the pre-feeding paradigm.

(B–E) Optogenetic silencing T1-DANs using T1-Gal4 (B and C) or T1′-Gal4 (D and E) abolished the PIFI effect but not the SIFI effect. n = 5–9. Student's t test for RFC. One-way ANOVA, Dunnett test for SI.

(F and G) Opto-inhibiting T1-DANs abolished the feeding inhibition induced by activating IPCs selectively for protein-containing foods, normal food (n = 5–7) and tryptone (n = 6–9), but not for sucrose (n = 6–7). The same set of data was used for the first and seventh columns in all three groups of experiments. One-way ANOVA, Dunnett test for the comparison within groups. See also Table S2 for two-way ANOVA comparison between groups.

n represents the number of trials. *$p < 0.05$, **$p < 0.01$, and ***$p < 0.001$. n.s. indicates no statistical significance. The data are shown in mean ± SEM.

See also Figure S4 and Table S3.

whereas SIFI was unaffected (Figures 4A–4C, S4A, and S4B). In contrast, silencing PPM3-DANs showed no influence on PIFI, and all these flies displayed normal PIFI compared to their parental groups when the light was off (Figures S4C and S4D). We additionally used T1′-Gal4 to silence T1 neurons and similarly found that the SI_T was significantly decreased in these flies, while SIFI was unaffected (Figures 4D, 4E, and S4E).

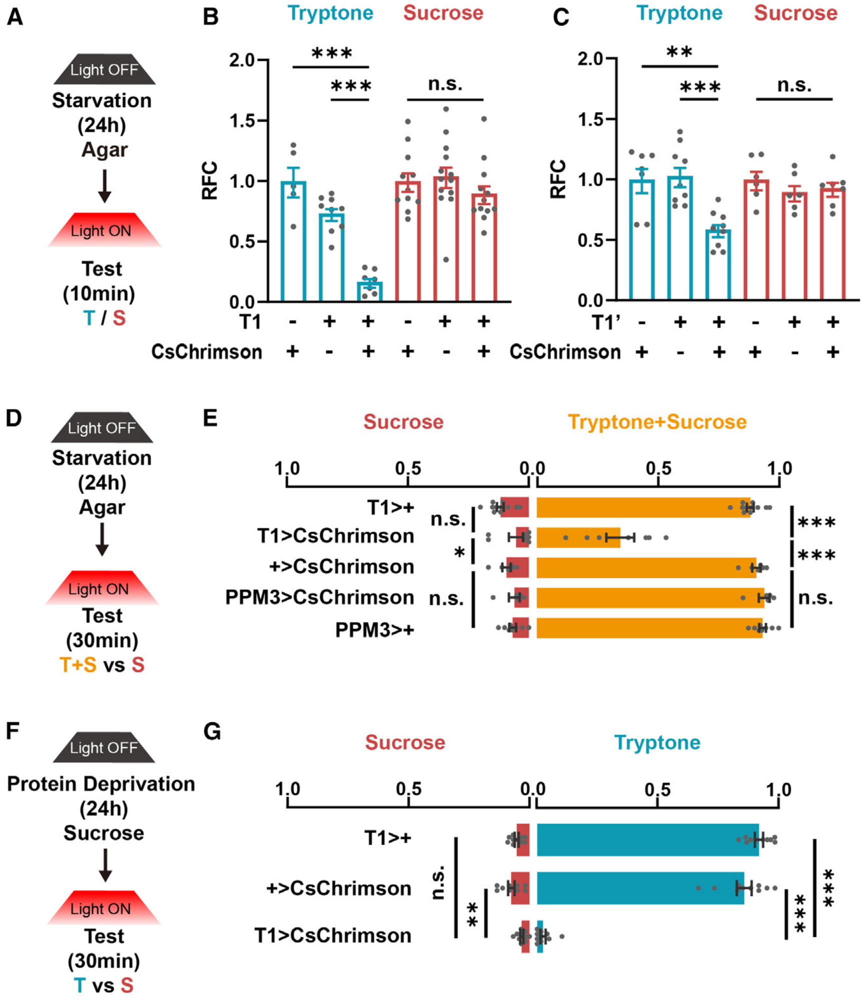

Figure 5. Activation of T1-DANs suppresses protein consumption

(A–C) Optogenetic activation of T1-DANs using two Gal4 stains both suppressed food consumption of tryptone but not sucrose. RFC, relative feeding consumption. n = 5–9 for tryptone and n = 11–13 for sucrose in (B). n = 7–9 for tryptone and n = 6–7 for sucrose in (C).

(D and E) In the two-choice assay, the choice ratio of protein-containing food was significantly reduced when activating T1-DANs, while it was unchanged when PPM3-DANs were activated. n = 5–11.

(F and G) Optogenetic activation of T1-DANs reduced protein choice in flies with 24 h protein deprivation. n = 10.

n represents the number of trials. One-way ANOVA, Dunnett test. $^{*}p < 0.05$, $^{**}p < 0.01$, and $^{***}p < 0.001$. n.s. indicates no statistical significance. The data are shown in mean ± SEM.

See also Figure S5.

We then tested whether silencing T1-DANs was sufficient to block the feeding inhibition induced by the insulin signal. As shown in Figures 4F and 4G, opto-activating IPCs suppressed the consumption of normal food in hungry flies. Remarkably, silencing T1-DANs completely abolished this suppression, although silencing T1-DANs alone did not affect the food intake in hungry flies. We further repeated this set of experiments using protein food and sugar food, respectively. The results showed that activating IPCs induced significant feeding inhibition in both types of foods, whereas silencing T1-DANs only blocked the group using protein food but not sugar food (Figures 4G, S4F, and S4G). We further performed a two-way ANOVA analysis. As shown in Table S2, in normal food and protein food experiments, activating IPCs together with inhibiting T1-DANs led to similar results to inhibiting T1-DANs only, whereas in sugar food experiments, activating IPCs together with inhibiting T1-DANs led to similar results to activating IPCs only. Therefore, these findings indicate that the pair of T1-DANs is one of the IPC-downstream neurons and selectively responds to the protein-satiety-induced insulin signal.

The next question was whether activating T1-DANs was sufficient to induce feeding inhibition. In fasted flies, opto-activating T1-DANs greatly reduced food consumption of protein but not sugar (Figures 5A–5C), while the control groups with light-OFF all showed normal food consumption (Figures S5A–S5C). In contrast, opto-activating PPM3-DANs did not affect protein consumption (Figure S5D). In agreement, in the two-choice assay,[51] the preference for protein-containing food was significantly decreased when T1-DANs were activated, whereas it was unaffected when PPM3-DANs were activated (Figures 5D and 5E). Moreover, activating T1-DANs also strongly suppressed the protein preference in flies with protein starvation for 24 h (Figures 5F, 5G, S5E, and S5F). Therefore, activating T1-DANs is sufficient to induce feeding inhibition specifically on protein-containing food.

T1-DANs convey the protein satiety signal to PB-LNs via dopamine signal

To determine the downstream neural circuit of T1-DANs, we expressed sytGFP to label the pre-synaptic sites of this pair of neurons. As shown in Figure 6A, this pre-synaptic signal was prominently localized in the projection terminals at the PB. We then performed syb:GRASP[52] experiments between T1-DANs and various types of PB neurons, including local neurons (LNs), eb-pb-gall (EPG), pb-eb-no (PEN), and pb-fb-no (PFN)[53–55] (Figure S6A). Strong GRASP signals were observed between T1-DANs and PB-LNs (Figure S6B). In agreement, we detected the postsynaptic DenMark signal of PB-LNs in the PB region (Figure 6B). Using the syb:GRASP approach,[52] we further examined the synaptic activity of T1-DANs to PB-LNs under different feeding conditions. Compared to the agar group, the synaptic activity significantly increased after protein consumption but not sugar consumption (Figure 6C). Therefore, these findings indicate that T1-DANs form a direct and dense

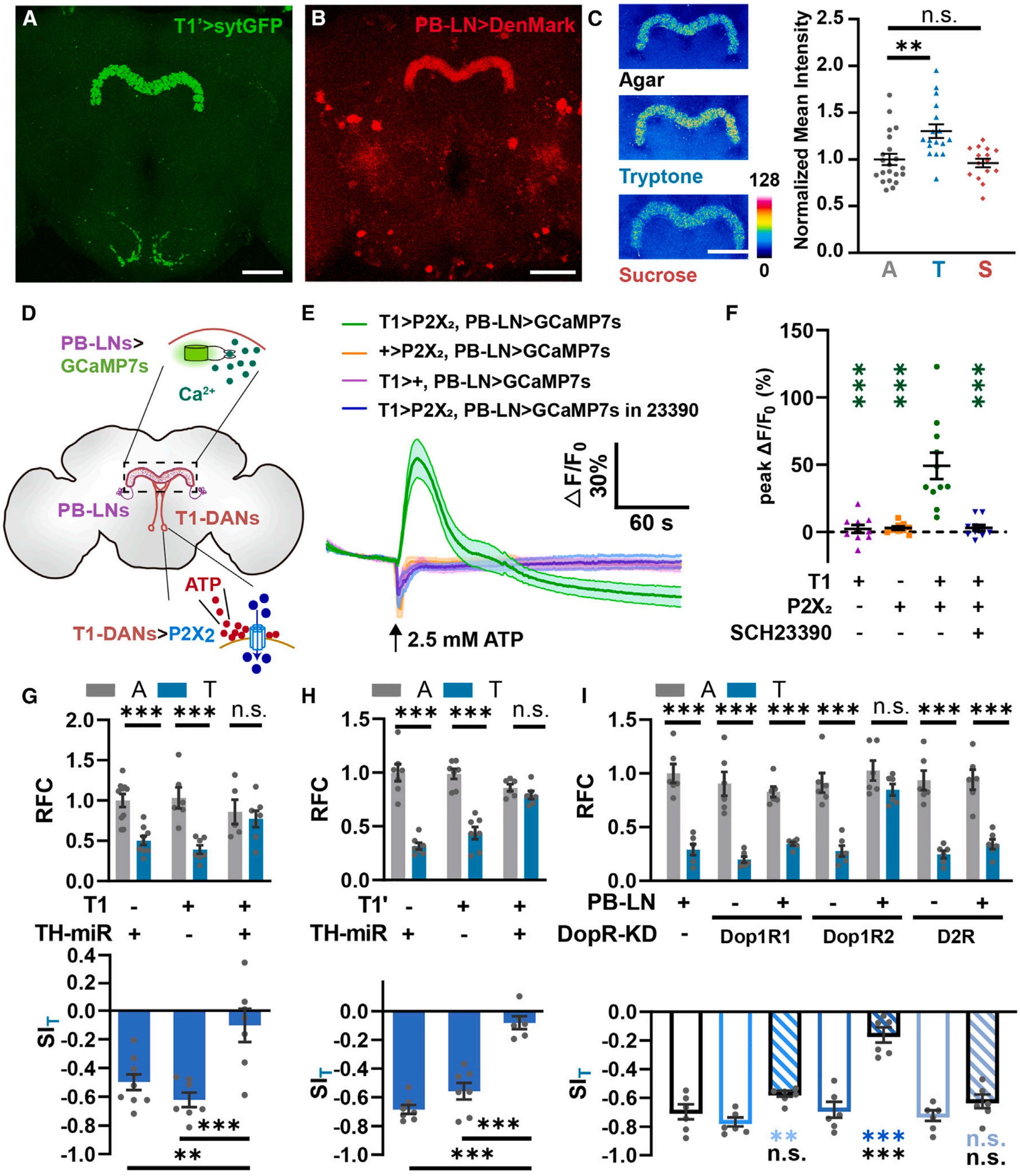

Figure 6. T1-DANs function through dopaminergic activation of PB-LNs

(A and B) The pre-synaptic signals of T1-DANs (A) and the postsynaptic sites of PB-LNs (B) are both concentrated in the brain region of PB. Scale bar, 50 μm.

(C) The syb:GRASP signals between T1-DANs and PB-LNs increased after protein consumption. Scale bar, 50 μm. The number of brains: n = 15–21.

(D–F) T1 activation induced a significant increase in calcium signals in PB-LNs. This induction was abolished when the antagonist of the dopamine D1-like receptor SCH23390 was supplied. The projection region of PB-LNs was selected as the ROI. The number of brains: n = 9–11.

(G and H) Knocking down TH in T1-DANs abolished PIFI effect. The number of trials: n = 5–10.

(I) KD of Dop1R2, but not Dop1R1 or D2R, in PB-LNs abolished PIFI effect. The number of trials: n = 6.

One-way ANOVA, Dunnett test in (C) and (F). Student's t test for RFC in (G)–(I). One-way ANOVA, Dunnett test for SI. *$p < 0.05$, **$p < 0.01$, and ***$p < 0.001$. n.s. indicates no statistical significance. The data are shown in mean ± SEM.

See also Figure S6.

CellPress
OPEN ACCESS

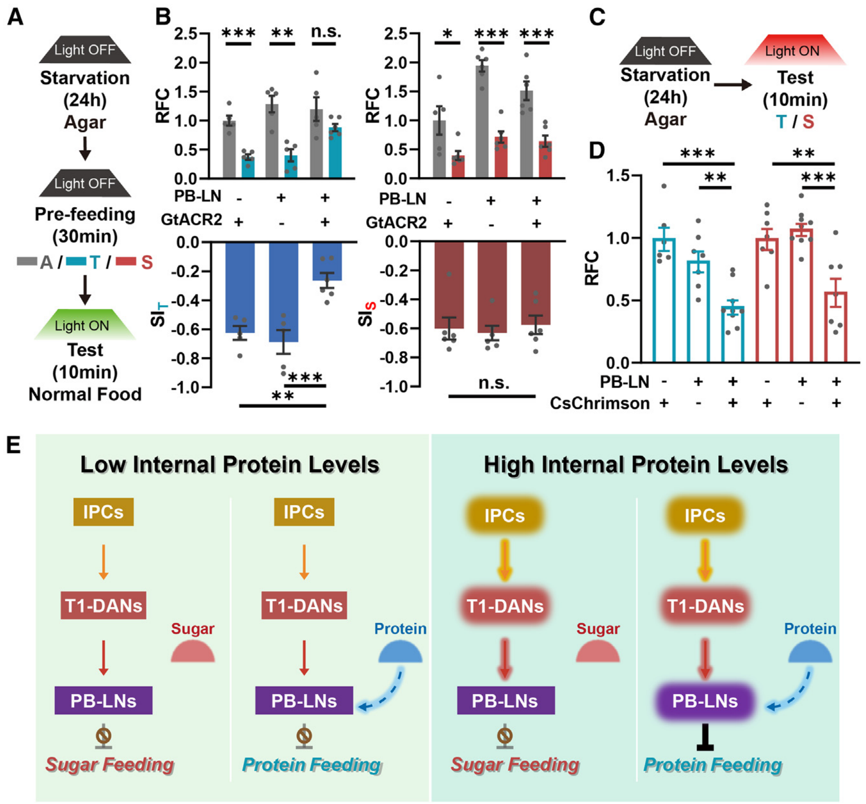

Figure 7. PB-LNs are required for PIFI

(A and B) Optogenetic silencing of PB-LNs suppressed PIFI but did not affect SIFI. n = 5–6. Student's t test for RFC. One-way ANOVA, Dunnett test for SI.

(C and D) Optogenetic activation of PB-LNs inhibited both tryptone and sucrose feeding. n = 6–9. One-way ANOVA, Dunnett test.

(E) The illustration of the neural circuits downstream of the insulin signal that is required for PIFI.

n represents the number of trials. $^*p < 0.05$, $^{**}p < 0.01$, and $^{***}p < 0.001$. n.s. indicates no statistical significance. The data are shown in mean ± SEM.

See also Figure S7.

connection with PB-LNs and that the synaptic transmission is enhanced specifically after protein intake.

We then tested for the functional connection between T1-DANs and PB-LNs. As shown in Figures 6D–6F, S6C, and S6D, activating T1-DANs using the ATP-P2X$_2$ system[47] triggered a strong increase in calcium levels of PB-LNs in the presence of TTX,[49] while genetic control groups lacking either Gal4 or P2X$_2$ did not show any response. Moreover, this increase was abolished when the dopamine D1-like receptor antagonist SCH 23390[56] was added into the bath, suggesting that T1-DANs activate PB-LNs through excitatory dopaminergic transmission. In the behavioral experiments, TH KD in T1-DANs resulted in defective PIFI (Figures 6G and 6H). Furthermore, knocking down the dopamine receptor Dop1R2 in PB-LNs also lead to a significant reduction in the SI_T (Figure 6I). Collectively, these results indicate that T1-DANs activate PB-LNs through Dop1R2 and that this dopaminergic activation of PB-LNs is required for PIFI.

We further examined whether PB-LNs are required for PIFI using the optogenetic approach. Consistent with the results of inhibiting T1-DANs, inhibiting PB-LNs strongly impaired PIFI, while SIFI was not affected (Figures 7A and 7B). However, different from activating T1-DANs, activating PB-LNs suppressed food consumption of both protein and sugar (Figures 7C, 7D, S7A, and S7B), suggesting that the information of protein-containing food is included in PB-LNs. As the control, inhibiting or activating these neurons did not affect the locomotion activity of flies (Figures S7C and S7D). Taken together, our findings reveal that the insulin signaling in T1-DANs represents high internal protein levels, which elicit a feeding termination signal in PB-LNs only when the food contains the protein nutrient.

DISCUSSION

The insulin signal in the brain has long been known to possess a strong suppressive effect on feeding behavior, while little is known about how it achieves this function.[57–59] Our study here reveals that in *Drosophila*, a brain-derived insulin signal directly activates a single pair of dopaminergic neurons, T1-DANs, and inhibits feeding through the downstream PB-LNs. Intriguingly, the insulin signal underlying this neural circuit elicits the suppressive effect on feeding only for protein food (Figure 7E). Therefore, we propose that insulin signals in the brain can encode satiety information in a nutrient-specific manner and precisely regulate feeding behavior.

Insulin signals in the brain play nutrient-specific roles in food satiation

Insulin is an ancient and conserved hormone serving as an important nutrient signal in regulating systematic development, metabolism, and behaviors.[60–63] In mammals, insulin levels change differentially after the consumption of different types of macronutrients.[3,4] In *Drosophila*, almost all known satiety signals are found to induce the release of DILPs or the activation of IPCs.[33,40,64–67] Intriguingly, the expression and/or release of DILPs is also found to be differentially regulated. For example, an earlier study showed that DILP2 is released in response to protein consumption, while DILP3 is released upon sugar consumption.[68] In addition, allatostatin A (AstA) is a satiety peptide selectively for carbohydrates, and silencing AstA neurons leads to an increase in DILP2 expression but a decrease in DILP3 expression.[69] Such differential regulation has also been found in studies of another two satiety factors, leucokinin (LK)[70] and *Drosophila* tackykinin (DTK).[71] Collectively, these studies suggest that the insulin system serves as the hub for food satiation of various nutrients, and the differential regulation of insulin peptides implies that the information of nutrient type is encoded in

the insulin system by different insulin/IGFs/ILPs, different insulin releasing cells, and/or different downstream circuits.

Although these insulin signals mentioned above appear to be nutrient specific, it has not been determined whether they participate in feeding regulation, let alone whether they regulate feeding in a nutrient-specific manner. Our study presented here shows that activating IPCs suppresses both protein and sugar intake, whereas silencing T1-DANs or PB-LNs blocks this feeding suppression only for protein food. These findings demonstrate that the insulin system mediates satiety signals of more than one type of nutrient and achieves nutrient-specific feeding regulation through specific downstream neural circuits. Intriguingly, a study in human and mouse demonstrated that insulin is secreted in response to the perfusion of amino acids (AAs) in fetal pancreas islets but to glucose in adult islets,[72] suggesting that the nutrient coding of the insulin system may dynamically change along development. Moreover, the DILP2 release was observed after protein feeding in adult female but not male flies,[40] implying a sexually dimorphic regulation of insulin signals. Future studies focusing on the diverse information encoded by brain-derived insulin signals will deepen the understanding of the CNS insulin system.

Nutrient sensing of internal protein levels and its roles in feeding regulation

As one of the macronutrients, protein is vital for animal development, health, and reproduction. To maintain the internal protein levels in the physiological range, both deficient and excess protein levels need to be monitored. General control nonderepressible 2 (GCN2) kinase can bind to uncharged tRNA and is activated when essential AAs (EAAs) are deficient.[73,74] At the behavioral level, GCN2 mutant mice exhibit a defect in the aversive response to EAA-imbalanced food.[75,76] In *Drosophila* larvae, the GCN2 signal functions in DL1-DANs to reject EAA-imbalanced food.[77] In adult flies, deficient EAAs increase the neural activity of WED-DANs, which are responsible for inducing feeding of protein food.[78] A recent study report that flies can sense the deficiency of a single type of AA, leucine, through the Sestrin-mTOR pathway.[79] In addition to the direct sensing in the brain, a gut peptide CNMamide (CNMa) indicates the low levels of EAAs in the gut and promotes protein feeding.[80] These signals monitor the lack of EAAs in the brain and gut for promoting consumption of more or better-balanced protein food.

For protein-specific satiety sensing, our previous work reported that a fat-body-expressed peptide, FIT, regulates PIFI through the insulin signaling.[40] In addition, two gut peptide hormones, diuretic hormone 31 (Dh31)[81] and CCHamide1 (CCHa1),[82] also selectively respond to protein consumption and trigger the switch from feeding to courtship and promote sleep, respectively. Based on the experimental setting, we suspect that Dh31 functions when the gut protein levels reach the lower boundary of the physiological range, CCHa1 functions when protein levels are high within the physiological range, and FIT functions when protein levels exceed the high boundary of the physiological range. Similar to FIT, the neural circuit of T1-DANs to PB-LNs identified in this study is related to insulin signaling and required for PIFI. Therefore, we propose that this circuit functions also when protein levels exceed the high boundary of the physiological range. Such nutrient-specific feeding inhibition enables effective and efficient protection from protein overeating.

T1-DAN-mediated protein satiety signal is subjected to further integration in PB

Our behavioral results show that silencing T1-DANs selectively blocks PIFI but not SIFI, and consistently, PB-LN silencing leads to the same results. These results indicate that the T1-PB pathway is specific for controlling overeating of protein but not sugar. However, different results were found when activating these neurons. Activating T1-DANs still selectively suppresses protein intake, whereas activating PB-LNs suppresses consumption of both protein and sugar foods. The loss of nutrient specificity in PB-LNs suggests that in addition to T1 input, other nutrient information is taken into account, presumably the existence of the protein nutrient in the food. PB-LNs exert an inhibitory role on feeding only when both the protein satiety signal (mediated by T1-DANs) and the protein-containing food signal (through an unknown pathway) are present.

PB is a component of the central complex (CC), which is the hub for behavioral regulation in adult fly. A recent study reported that another component of the CC, the fan-shaped body (FB), plays an essential role in sugar sensing and feeding preference.[83] Thus, the nutrient information of protein and sugar are processed separately in the CC. There are multiple types of neurons linking different parts of the CC, e.g., the PFN and pb-fb-idfp (PFI) linking FB and PB,[54] raising the possibility of further integration of these two types of nutrient signals. The CC plays various roles in behavior regulation, including locomotion, navigation, sleep, and social behavior.[54,84–87] The protein satiety signal may also participate in the modulation of these behaviors. Further investigations on its roles in these behaviors will deepen our understanding of how nutrient signals coordinately regulate multiple behavior modules.

Limitations of the study

Our findings in this study uncover that the T1-PB circuit, as one of the downstream of IPCs, selectively adopts the insulin-mediated protein-specific satiety signal. However, it remains undetermined whether there is another downstream circuit that selectively adopts the sugar-specific satiety signal. It would be intriguing to know how, in response to food intake of different types of nutrients, regulatory signals stimulate IPCs in different ways, how DILPs in the IPCs encode different nutrient-specific satiety, and how different downstream circuits selectively adopt these signals. In addition, we speculate that in addition to the T1-mediated protein satiety signal, a signal representing protein food is required to activate PB-LNs and elicit feeding inhibition. It would be valuable to find out the neural input of this food nutrient signal and the circuit mechanism for integrating these two signals.

STAR★METHODS

Detailed methods are provided in the online version of this paper and include the following:

- KEY RESOURCES TABLE
- RESOURCE AVAILABILITY

CellPress
OPEN ACCESS

- Lead contact
- Materials availability
- Data and code availability
- EXPERIMENTAL MODEL AND STUDY PARTICIPANT DETAILS
  - Fly strains and cultivation
- METHOD DETAILS
  - Behavior assays
  - Imaging
  - ELISA
- QUANTIFICATION AND STATISTICAL ANALYSIS

SUPPLEMENTAL INFORMATION

Supplemental information can be found online at https://doi.org/10.1016/j.celrep.2024.114282.

ACKNOWLEDGMENTS

We thank Professors Ronald L. Davis and Zhefeng Gong, as well as the Bloomington *Drosophila* Stock Center and Vienna *Drosophila* Research Center, for gifting fly strains. We thank Professor Wei Zhang, Dr. Mingming Zhou, Dr. Junyu Zhao, Jing Guo, Yuanyuan Zhao, and Mingxin Liu for helpful discussions and technical support. This work was supported by the National Key R&D Program of China (2019YFA0802402), the Space Application Project of the Space Station (YYWT-0801-EXP-13), the National Natural Science Foundation of China (grants 32211540388, 32061143011, 31970947, and 31730045), the Chinese Academy of Sciences Interdisciplinary Innovation Team to Y.L., and Bill and Melinda Gates Foundation (OPP1119434) to Y.Z.

AUTHOR CONTRIBUTIONS

Y.L., X.B., and X.L. conceived this project and designed experiments. Y.L. secured funding. X.L., X.B., and X.W. performed the behavioral experiments and analyzed the data. Y.Y., X.L., H.T., and Y.C. performed the functional calcium imaging and immunostaining experiments. X.L. and Q.L. generated the transgenic fly strains. Y.L., X.L., Y.Y., and X.W. prepared the figures and wrote the manuscript. M.N.W. and Y.Z. provided expertise and feedback. All authors read and approved the manuscript.

DECLARATION OF INTERESTS

The authors declare no competing interests.

Received: August 25, 2023
Revised: April 8, 2024
Accepted: May 10, 2024
Published: May 24, 2024

REFERENCES

1. Dimitriadis, G.D., Maratou, E., Kountouri, A., Board, M., and Lambadiari, V. (2021). Regulation of Postabsorptive and Postprandial Glucose Metabolism by Insulin-Dependent and Insulin-Independent Mechanisms: An Integrative Approach. Nutrients *13*, 159. https://doi.org/10.3390/nu13010159.
2. Flint, A., Gregersen, N.T., Gluud, L.L., Møller, B.K., Raben, A., Tetens, I., Verdich, C., and Astrup, A. (2007). Associations between postprandial insulin and blood glucose responses, appetite sensations and energy intake in normal weight and overweight individuals: a meta-analysis of test meal studies. Br. J. Nutr. *98*, 17–25. https://doi.org/10.1017/S000711450768297X.
3. Gerozissis, K., Rouch, C., Nicolaidis, S., and Orosco, M. (1999). Brain insulin response to feeding in the rat is both macronutrient and area specific. Physiol. Behav. *66*, 271–275. https://doi.org/10.1016/s0031-9384(99)00061-x.
4. Gerozissis, K., Orosco, M., Rouch, C., and Nicolaidis, S. (1997). Insulin responses to a fat meal in hypothalamic microdialysates and plasma. Physiol. Behav. *62*, 767–772. https://doi.org/10.1016/s0031-9384(97)00195-9.
5. Banks, W.A., Jaspan, J.B., Huang, W., and Kastin, A.J. (1997). Transport of insulin across the blood-brain barrier: saturability at euglycemic doses of insulin. Peptides *18*, 1423–1429. https://doi.org/10.1016/s0196-9781(97)00231-3.
6. Margolis, R.U., and Altszuler, N. (1967). Insulin in the cerebrospinal fluid. Nature *215*, 1375–1376. https://doi.org/10.1038/2151375a0.
7. Kondo, M. (2023). Molecular Mechanisms of Exercise-induced Hippocampal Neurogenesis and Antidepressant Effects. JMA J *6*, 114–119. https://doi.org/10.31662/jmaj.2023-0010.
8. Lee, J., Kim, K., Cho, J.H., Bae, J.Y., O'Leary, T.P., Johnson, J.D., Bae, Y.C., and Kim, E.K. (2020). Insulin synthesized in the paraventricular nucleus of the hypothalamus regulates pituitary growth hormone production. JCI Insight *5*, e135412. https://doi.org/10.1172/jci.insight.135412.
9. Molnar, G., Farago, N., Kocsis, A.K., Rozsa, M., Lovas, S., Boldog, E., Baldi, R., Csajbok, E., Gardi, J., Puskas, L.G., and Tamas, G. (2014). GABAergic neurogliaform cells represent local sources of insulin in the cerebral cortex. J. Neurosci. *34*, 1133–1137. https://doi.org/10.1523/JNEUROSCI.4082-13.2014.
10. Pitt, J., Wilcox, K.C., Tortelli, V., Diniz, L.P., Oliveira, M.S., Dobbins, C., Yu, X.W., Nandamuri, S., Gomes, F.C.A., DiNunno, N., et al. (2017). Neuroprotective astrocyte-derived insulin/insulin-like growth factor 1 stimulates endocytic processing and extracellular release of neuron-bound Abeta oligomers. Mol. Biol. Cell *28*, 2623–2636. https://doi.org/10.1091/mbc.E17-06-0416.
11. Siddle, K., Soos, M.A., Field, C.E., and Navé, B.T. (1994). Hybrid and atypical insulin/insulin-like growth factor I receptors. Horm. Res. *41*, 56–65. https://doi.org/10.1159/000183962.
12. Pandini, G., Frasca, F., Mineo, R., Sciacca, L., Vigneri, R., and Belfiore, A. (2002). Insulin/insulin-like growth factor I hybrid receptors have different biological characteristics depending on the insulin receptor isoform involved. J. Biol. Chem. *277*, 39684–39695. https://doi.org/10.1074/jbc.M202766200.
13. Salmon, W.D., Jr., and Daughaday, W.H. (1957). A hormonally controlled serum factor which stimulates sulfate incorporation by cartilage in vitro. J. Lab. Clin. Med. *49*, 825–836.
14. Annunziata, M., Granata, R., and Ghigo, E. (2011). The IGF system. Acta Diabetol. *48*, 1–9.
15. Bayne, M.L., Applebaum, J., Underwood, D., Chicchi, G.G., Green, B.G., Hayes, N.S., and Cascieri, M.A. (1989). The C region of human insulin-like growth factor (IGF) I is required for high affinity binding to the type 1 IGF receptor. J. Biol. Chem. *264*, 11004–11008.
16. Denley, A., Bonython, E.R., Booker, G.W., Cosgrove, L.J., Forbes, B.E., Ward, C.W., and Wallace, J.C. (2004). Structural determinants for high-affinity binding of insulin-like growth factor II to insulin receptor (IR)-A, the exon 11 minus isoform of the IR. Mol. Endocrinol. *18*, 2502–2512. https://doi.org/10.1210/me.2004-0183.
17. Umezaki, Y., Hayley, S.E., Chu, M.L., Seo, H.W., Shah, P., and Hamada, F.N. (2018). Feeding-State-Dependent Modulation of Temperature Preference Requires Insulin Signaling in Drosophila Warm-Sensing Neurons. Curr. Biol. *28*, 779–787.e3. https://doi.org/10.1016/j.cub.2018.01.060.
18. Wigby, S., Slack, C., Grönke, S., Martinez, P., Calboli, F.C.F., Chapman, T., and Partridge, L. (2011). Insulin signalling regulates remating in female Drosophila. Proc. Biol. Sci. *278*, 424–431. https://doi.org/10.1098/rspb.2010.1390.
19. Benedict, C., Hallschmid, M., Hatke, A., Schultes, B., Fehm, H.L., Born, J., and Kern, W. (2004). Intranasal insulin improves memory in humans. Psychoneuroendocrinology *29*, 1326–1334. https://doi.org/10.1016/j.psyneuen.2004.04.003.
20. Kern, W., Peters, A., Fruehwald-Schultes, B., Deininger, E., Born, J., and Fehm, H.L. (2001). Improving influence of insulin on cognitive functions

in humans. Neuroendocrinology *74*, 270–280. https://doi.org/10.1159/000054694.

21. Woods, S.C., Lotter, E.C., Mckay, L.D., and Porte, D. (1979). Chronic Intracerebroventricular Infusion of Insulin Reduces Food-Intake and Body-Weight of Baboons. Nature *282*, 503–505.
22. Florant, G.L., Singer, L., Scheurink, A.J., Park, C.R., Richardson, R.D., and Woods, S.C. (1991). Intraventricular Insulin Reduces Food-Intake and Body-Weight of Marmots during the Summer Feeding Period. Physiol. Behav. *49*, 335–338.
23. Brief, D.J., and Davis, J.D. (1984). Reduction of Food-Intake and Body-Weight by Chronic Intraventricular Insulin Infusion. Brain Res. Bull. *12*, 571–575.
24. Vasselli, J.R., Pi-Sunyer, F.X., Wall, D.G., John, C.S., Chapman, C.D., and Currie, P.J. (2017). Central effects of insulin detemir on feeding, body weight, and metabolism in rats. Am. J. Physiol. Endocrinol. Metab. *313*, E613–E621.
25. Bruning, J.C., Gautam, D., Burks, D.J., Gillette, J., Schubert, M., Orban, P.C., Klein, R., Krone, W., Muller-Wieland, D., and Kahn, C.R. (2000). Role of brain insulin receptor in control of body weight and reproduction. Science *289*, 2122–2125. https://doi.org/10.1126/science.289.5487.2122.
26. Dodd, G.T., Kim, S.J., Mequinion, M., Xirouchaki, C.E., Bruning, J.C., Andrews, Z.B., and Tiganis, T. (2021). Insulin signaling in AgRP neurons regulates meal size to limit glucose excursions and insulin resistance. Sci. Adv. *7*, eabf4100. https://doi.org/10.1126/sciadv.abf4100.
27. Eerola, K., Longo, F., Reinbothe, T.M., Richard, J.E., Shevchouk, O.T., López-Ferreras, L., Mishra, D., Asker, M., Tolö, J., Miranda, C., et al. (2022). Hindbrain insulin controls feeding behavior. Mol. Metab. *66*, 101614. https://doi.org/10.1016/j.molmet.2022.101614.
28. Zhan, Y.P., Liu, L., and Zhu, Y. (2016). Taotie neurons regulate appetite in Drosophila. Nat. Commun. *7*, 13633. https://doi.org/10.1038/ncomms13633.
29. Hong, S.H., Lee, K.S., Kwak, S.J., Kim, A.K., Bai, H., Jung, M.S., Kwon, O.Y., Song, W.J., Tatar, M., and Yu, K. (2012). Minibrain/Dyrk1a Regulates Food Intake through the Sir2-FOXO-sNPF/NPY Pathway in Drosophila and Mammals. PLoS Genet. *8*, e1002857. https://doi.org/10.1371/journal.pgen.1002857.
30. Root, C.M., Ko, K.I., Jafari, A., and Wang, J.W. (2011). Presynaptic facilitation by neuropeptide signaling mediates odor-driven food search. Cell *145*, 133–144. https://doi.org/10.1016/j.cell.2011.02.008.
31. Wu, Q., Zhang, Y., Xu, J., and Shen, P. (2005). Regulation of hunger-driven behaviors by neural ribosomal S6 kinase in Drosophila. Proc. Natl. Acad. Sci. USA *102*, 13289–13294. https://doi.org/10.1073/pnas.0501914102.
32. Wu, Q., Zhao, Z., and Shen, P. (2005). Regulation of aversion to noxious food by Drosophila neuropeptide Y- and insulin-like systems. Nat. Neurosci. *8*, 1350–1355. https://doi.org/10.1038/nn1540.
33. Wang, P., Jia, Y., Liu, T., Jan, Y.N., and Zhang, W. (2020). Visceral Mechano-sensing Neurons Control Drosophila Feeding by Using Piezo as a Sensor. Neuron *108*, 640–650.e4. https://doi.org/10.1016/j.neuron.2020.08.017.
34. Zhao, X.L., and Campos, A.R. (2012). Insulin signalling in mushroom body neurons regulates feeding behaviour in Drosophila larvae. J. Exp. Biol. *215*, 2696–2702. https://doi.org/10.1242/jeb.066969.
35. Liu, Y., Luo, J., Carlsson, M.A., and Nässel, D.R. (2015). Serotonin and insulin-like peptides modulate leucokinin-producing neurons that affect feeding and water homeostasis in Drosophila. J. Comp. Neurol. *523*, 1840–1863. https://doi.org/10.1002/cne.23768.
36. González Segarra, A.J., Pontes, G., Jourjine, N., Del Toro, A., and Scott, K. (2023). Hunger- and thirst-sensing neurons modulate a neuroendocrine network to coordinate sugar and water ingestion. Elife *12*, RP88143. https://doi.org/10.7554/eLife.88143.
37. Sudhakar, S.R., Pathak, H., Rehman, N., Fernandes, J., Vishnu, S., and Varghese, J. (2020). Insulin signalling elicits hunger-induced feeding in Drosophila. Dev. Biol. *459*, 87–99. https://doi.org/10.1016/j.ydbio.2019.11.013.
38. Claessens, M., Saris, W.H.M., and van Baak, M.A. (2008). Glucagon and insulin responses after ingestion of different amounts of intact and hydrolysed proteins. Br. J. Nutr. *100*, 61–69. https://doi.org/10.1017/S0007114507886314.
39. Yoshizane, C., Mizote, A., Yamada, M., Arai, N., Arai, S., Maruta, K., Mitsuzumi, H., Ariyasu, T., Ushio, S., and Fukuda, S. (2017). Glycemic, insulinemic and incretin responses after oral trehalose ingestion in healthy subjects. Nutr. J. *16*, 9. https://doi.org/10.1186/s12937-017-0233-x.
40. Sun, J., Liu, C., Bai, X., Li, X., Li, J., Zhang, Z., Zhang, Y., Guo, J., and Li, Y. (2017). Drosophila FIT is a protein-specific satiety hormone essential for feeding control. Nat. Commun. *8*, 14161. https://doi.org/10.1038/ncomms14161.
41. A, P. (2004). FlyBase Reference Report: Parks, 2004.5.12, InR Constructs and Insertions.
42. Broughton, S., Alic, N., Slack, C., Bass, T., Ikeya, T., Vinti, G., Tommasi, A.M., Driege, Y., Hafen, E., and Partridge, L. (2008). Reduction of DILP2 in Drosophila triages a metabolic phenotype from lifespan revealing redundancy and compensation among DILPs. PLoS One *3*, e3721. https://doi.org/10.1371/journal.pone.0003721.
43. Luan, H., Diao, F., Scott, R.L., and White, B.H. (2020). The Drosophila Split Gal4 System for Neural Circuit Mapping. Front. Neural Circuits *14*, 603397. https://doi.org/10.3389/fncir.2020.603397.
44. Fosque, B.F., Sun, Y., Dana, H., Yang, C.T., Ohyama, T., Tadross, M.R., Patel, R., Zlatic, M., Kim, D.S., Ahrens, M.B., et al. (2015). Labeling of active neural circuits in vivo with designed calcium integrators. Science *347*, 755–760. https://doi.org/10.1126/science.1260922.
45. Park, S., Alfa, R.W., Topper, S.M., Kim, G.E.S., Kockel, L., and Kim, S.K. (2014). A Genetic Strategy to Measure Circulating Drosophila Insulin Reveals Genes Regulating Insulin Production and Secretion. PLoS Genet. *10*, e1004555. https://doi.org/10.1371/journal.pgen.1004555.
46. Gordon, M.D., and Scott, K. (2009). Motor control in a Drosophila taste circuit. Neuron *61*, 373–384. https://doi.org/10.1016/j.neuron.2008.12.033.
47. Lima, S.Q., and Miesenböck, G. (2005). Remote control of behavior through genetically targeted photostimulation of neurons. Cell *121*, 141–152. https://doi.org/10.1016/j.cell.2005.02.004.
48. Yao, Z., Macara, A.M., Lelito, K.R., Minosyan, T.Y., and Shafer, O.T. (2012). Analysis of functional neuronal connectivity in the Drosophila brain. J. Neurophysiol. *108*, 684–696. https://doi.org/10.1152/jn.00110.2012.
49. Lee, C.H., and Ruben, P.C. (2008). Interaction between voltage-gated sodium channels and the neurotoxin, tetrodotoxin. Channels *2*, 407–412. https://doi.org/10.4161/chan.2.6.7429.
50. Mauss, A.S., Busch, C., and Borst, A. (2017). Optogenetic Neuronal Silencing in Drosophila during Visual Processing. Sci. Rep. *7*, 13823. https://doi.org/10.1038/s41598-017-14076-7.
51. Ribeiro, C., and Dickson, B.J. (2010). Sex Peptide Receptor and Neuronal TOR/S6K Signaling Modulate Nutrient Balancing in Drosophila. Curr. Biol. *20*, 1000–1005. https://doi.org/10.1016/j.cub.2010.03.061.
52. Macpherson, L.J., Zaharieva, E.E., Kearney, P.J., Alpert, M.H., Lin, T.Y., Turan, Z., Lee, C.H., and Gallio, M. (2015). Dynamic labelling of neural connections in multiple colours by trans-synaptic fluorescence complementation. Nat. Commun. *6*, 10024. https://doi.org/10.1038/ncomms10024.
53. Hanesch, U., Fischbach, K.F., and Heisenberg, M. (1989). Neuronal architecture of the central complex in Drosophila melanogaster. Cell Tissue Res. *257*, 343–366. https://doi.org/10.1007/BF00261838.
54. Lin, C.Y., Chuang, C.C., Hua, T.E., Chen, C.C., Dickson, B.J., Greenspan, R.J., and Chiang, A.S. (2013). A comprehensive wiring diagram of the protocerebral bridge for visual information processing in the Drosophila brain. Cell Rep. *3*, 1739–1753. https://doi.org/10.1016/j.celrep.2013.04.022.
55. Wolff, T., Iyer, N.A., and Rubin, G.M. (2015). Neuroarchitecture and neuroanatomy of the Drosophila central complex: A GAL4-based dissection of

protocerebral bridge neurons and circuits. J. Comp. Neurol. *523*, 997–1037. https://doi.org/10.1002/cne.23705.

56. Bourne, J.A. (2001). SCH 23390: the first selective dopamine D1-like receptor antagonist. CNS Drug Rev. *7*, 399–414. https://doi.org/10.1111/j.1527-3458.2001.tb00207.x.

57. Ferrario, C.R., and Finnell, J.E. (2023). Beyond the hypothalamus: roles for insulin as a regulator of neurotransmission, motivation, and feeding. Neuropsychopharmacology *48*, 232–233. https://doi.org/10.1038/s41386-022-01398-y.

58. Chen, W., Cai, W., Hoover, B., and Kahn, C.R. (2022). Insulin action in the brain: cell types, circuits, and diseases. Trends Neurosci. *45*, 384–400. https://doi.org/10.1016/j.tins.2022.03.001.

59. Dakic, T., Jevdjovic, T., Lakic, I., Ruzicic, A., Jasnic, N., Djurasevic, S., Djordjevic, J., and Vujovic, P. (2023). The Expression of Insulin in the Central Nervous System: What Have We Learned So Far? Int. J. Mol. Sci. *24*, 6586. https://doi.org/10.3390/ijms24076586.

60. Straus, D.S. (1984). Growth-stimulatory actions of insulin in vitro and in vivo. Endocr. Rev. *5*, 356–369. https://doi.org/10.1210/edrv-5-2-356.

61. Sims, E.K., Carr, A.L.J., Oram, R.A., DiMeglio, L.A., and Evans-Molina, C. (2021). 100 years of insulin: celebrating the past, present and future of diabetes therapy. Nat. Med. *27*, 1154–1164. https://doi.org/10.1038/s41591-021-01418-2.

62. Speakman, J.R., and Hall, K.D. (2021). Carbohydrates, insulin, and obesity. Science *372*, 577–578. https://doi.org/10.1126/science.aav0448.

63. Banks, W.A., Owen, J.B., and Erickson, M.A. (2012). Insulin in the brain: There and back again. Pharmacol. Ther. *136*, 82–93. https://doi.org/10.1016/j.pharmthera.2012.07.006.

64. Oh, Y., Lai, J.S.Y., Mills, H.J., Erdjument-Bromage, H., Giammarinaro, B., Saadipour, K., Wang, J.G., Abu, F., Neubert, T.A., and Suh, G.S.B. (2019). A glucose-sensing neuron pair regulates insulin and glucagon in Drosophila. Nature *574*, 559–564. https://doi.org/10.1038/s41586-019-1675-4.

65. Rajan, A., and Perrimon, N. (2012). Drosophila Cytokine Unpaired 2 Regulates Physiological Homeostasis by Remotely Controlling Insulin Secretion. Cell *151*, 123–137. https://doi.org/10.1016/j.cell.2012.08.019.

66. Yao, Z., and Scott, K. (2022). Serotonergic neurons translate taste detection into internal nutrient regulation. Neuron *110*, 1036–1050.e7. https://doi.org/10.1016/j.neuron.2021.12.028.

67. Semaniuk, U., Strilbytska, O., Malinovska, K., Storey, K.B., Vaiserman, A., Lushchak, V., and Lushchak, O. (2021). Factors that regulate expression patterns of insulin-like peptides and their association with physiological and metabolic traits in Drosophila. Insect Biochem. Mol. Biol. *135*, 103609. https://doi.org/10.1016/j.ibmb.2021.103609.

68. Kim, J., and Neufeld, T.P. (2015). Dietary sugar promotes systemic TOR activation in Drosophila through AKH-dependent selective secretion of Dilp3. Nat. Commun. *6*, 6846. https://doi.org/10.1038/ncomms7846.

69. Hentze, J.L., Carlsson, M.A., Kondo, S., Nässel, D.R., and Rewitz, K.F. (2015). The Neuropeptide Allatostatin A Regulates Metabolism and Feeding Decisions in Drosophila. Sci. Rep. *5*, 11680. https://doi.org/10.1038/srep11680.

70. Zandawala, M., Yurgel, M.E., Texada, M.J., Liao, S., Rewitz, K.F., Keene, A.C., and Nässel, D.R. (2018). Modulation of Drosophila post-feeding physiology and behavior by the neuropeptide leucokinin. PLoS Genet. *14*, e1007767. https://doi.org/10.1371/journal.pgen.1007767.

71. Birse, R.T., Söderberg, J.A.E., Luo, J., Winther, A.M.E., and Nässel, D.R. (2011). Regulation of insulin-producing cells in the adult Drosophila brain via the tachykinin peptide receptor DTKR. J. Exp. Biol. *214*, 4201–4208. https://doi.org/10.1242/jeb.062091.

72. Helman, A., Cangelosi, A.L., Davis, J.C., Pham, Q., Rothman, A., Faust, A.L., Straubhaar, J.R., Sabatini, D.M., and Melton, D.A. (2020). A Nutrient-Sensing Transition at Birth Triggers Glucose-Responsive Insulin Secretion. Cell Metab. *31*, 1004–1016.e5. https://doi.org/10.1016/j.cmet.2020.04.004.

73. Wek, S.A., Zhu, S., and Wek, R.C. (1995). The histidyl-tRNA synthetase-related sequence in the eIF-2 alpha protein kinase GCN2 interacts with tRNA and is required for activation in response to starvation for different amino acids. Mol. Cell Biol. *15*, 4497–4506. https://doi.org/10.1128/MCB.15.8.4497.

74. Dong, J., Qiu, H., Garcia-Barrio, M., Anderson, J., and Hinnebusch, A.G. (2000). Uncharged tRNA activates GCN2 by displacing the protein kinase moiety from a bipartite tRNA-binding domain. Mol. Cell *6*, 269–279. https://doi.org/10.1016/s1097-2765(00)00028-9.

75. Hao, S., Sharp, J.W., Ross-Inta, C.M., McDaniel, B.J., Anthony, T.G., Wek, R.C., Cavener, D.R., McGrath, B.C., Rudell, J.B., Koehnle, T.J., and Gietzen, D.W. (2005). Uncharged tRNA and sensing of amino acid deficiency in mammalian piriform cortex. Science *307*, 1776–1778. https://doi.org/10.1126/science.1104882.

76. Maurin, A.C., Jousse, C., Averous, J., Parry, L., Bruhat, A., Cherasse, Y., Zeng, H., Zhang, Y., Harding, H.P., Ron, D., and Fafournoux, P. (2005). The GCN2 kinase biases feeding behavior to maintain amino acid homeostasis in omnivores. Cell Metab. *1*, 273–277. https://doi.org/10.1016/j.cmet.2005.03.004.

77. Bjordal, M., Arquier, N., Kniazeff, J., Pin, J.P., and Léopold, P. (2014). Sensing of Amino Acids in a Dopaminergic Circuitry Promotes Rejection of an Incomplete Diet in Drosophila. Cell *156*, 510–521. https://doi.org/10.1016/j.cell.2013.12.024.

78. Liu, Q., Tabuchi, M., Liu, S., Kodama, L., Horiuchi, W., Daniels, J., Chiu, L., Baldoni, D., and Wu, M.N. (2017). Branch-specific plasticity of a bifunctional dopamine circuit encodes protein hunger. Science *356*, 534–539. https://doi.org/10.1126/science.aal3245.

79. Gu, X., Jouandin, P., Lalgudi, P.V., Binari, R., Valenstein, M.L., Reid, M.A., Allen, A.E., Kamitaki, N., Locasale, J.W., Perrimon, N., and Sabatini, D.M. (2022). Sestrin mediates detection of and adaptation to low-leucine diets in Drosophila. Nature *608*, 209–216. https://doi.org/10.1038/s41586-022-04960-2.

80. Kim, B., Kanai, M.I., Oh, Y., Kyung, M., Kim, E.K., Jang, I.H., Lee, J.H., Kim, S.G., Suh, G.S.B., and Lee, W.J. (2021). Response of the microbiome-gut-brain axis in Drosophila to amino acid deficit. Nature *593*, 570–574. https://doi.org/10.1038/s41586-021-03522-2.

81. Lin, H.H., Kuang, M.C., Hossain, I., Xuan, Y., Beebe, L., Shepherd, A.K., Rolandi, M., and Wang, J.W. (2022). A nutrient-specific gut hormone arbitrates between courtship and feeding. Nature *602*, 632–638. https://doi.org/10.1038/s41586-022-04408-7.

82. Titos, I., Juginovic, A., Vaccaro, A., Nambara, K., Gorelik, P., Mazor, O., and Rogulja, D. (2023). A gut-secreted peptide suppresses arousability from sleep. Cell *186*, 1382–1397.e1321. https://doi.org/10.1016/j.cell.2023.02.022.

83. Musso, P.Y., Junca, P., and Gordon, M.D. (2021). A neural circuit linking two sugar sensors regulates satiety-dependent fructose drive in Drosophila. Sci. Adv. *7*, eabj0186. https://doi.org/10.1126/sciadv.abj0186.

84. Flores-Valle, A., Goncalves, P.J., and Seelig, J.D. (2021). Integration of sleep homeostasis and navigation in Drosophila. PLoS Comput. Biol. *17*.

85. Goulard, R., Buehlmann, C., Niven, J.E., Graham, P., and Webb, B. (2021). A unified mechanism for innate and learned visual landmark guidance in the insect central complex. PLoS Comput. Biol. *17*, e1009383.

86. Popov, A.V., Peresleni, A.I., Ozerskiĭ, P.V., Shchekanov, E.E., and Savvateeva-Popova, E.V. (2003). [Role of the protocerebral bridge in the central complex of Drosophila melanogaster brain in the control of courtship behavior and sound production of males]. Zh. Evol. Biokhim. Fiziol. *39*, 530–539.

87. Strauss, R., Hanesch, U., Kinkelin, M., Wolf, R., and Heisenberg, M. (1992). No-bridge of Drosophila melanogaster: portrait of a structural brain mutant of the central complex. J. Neurogenet. *8*, 125–155. https://doi.org/10.3109/01677069209083444.

88. Schneider, C.A., Rasband, W.S., and Eliceiri, K.W. (2012). NIH Image to ImageJ: 25 years of image analysis. Nat. Methods *9*, 671–675. https://doi.org/10.1038/nmeth.2089.

89. Xie, T., Ho, M.C.W., Liu, Q., Horiuchi, W., Lin, C.-C., Task, D., Luan, H., White, B.H., Potter, C.J., and Wu, M.N. (2018). A Genetic Toolkit for Dissecting Dopamine Circuit Function in Drosophila. Cell Rep. *23*, 652–665. https://doi.org/10.1016/j.celrep.2018.03.068.

90. Berry, J.A., Cervantes-Sandoval, I., Nicholas, E.P., and Davis, R.L. (2012). Dopamine Is Required for Learning and Forgetting in Drosophila. Neuron *74*, 530–542. https://doi.org/10.1016/j.neuron.2012.04.007.

91. Li, Q., and Gong, Z. (2015). Cold-sensing regulates Drosophila growth through insulin-producing cells. Nat. Commun. *6*, 10083. https://doi.org/10.1038/ncomms10083.

92. Romero, E., Cha, G.-H., Verstreken, P., Ly, C.V., Hughes, R.E., Bellen, H.J., and Botas, J. (2008). Suppression of Neurodegeneration and Increased Neurotransmission Caused by Expanded Full-Length Huntingtin Accumulating in the Cytoplasm. Neuron *57*, 27–40. https://doi.org/10.1016/j.neuron.2007.11.025.

93. Talay, M., Richman, E.B., Snell, N.J., Hartmann, G.G., Fisher, J.D., Sorkaç, A., Santoyo, J.F., Chou-Freed, C., Nair, N., Johnson, M., et al. (2017). Transsynaptic Mapping of Second-Order Taste Neurons in Flies by trans-Tango. Neuron *96*, 783–795.e4. https://doi.org/10.1016/j.neuron.2017.10.011.

94. Wilson, R.I., Turner, G.C., and Laurent, G. (2004). Transformation of Olfactory Representations in the *Drosophila* Antennal Lobe. Science *303*, 366–370. https://doi.org/10.1126/science.1090782.

95. Tennessen, J.M., Barry, W.E., Cox, J., and Thummel, C.S. (2014). Methods for studying metabolism in Drosophila. Methods *68*, 105–115. https://doi.org/10.1016/j.ymeth.2014.02.034.

STAR★METHODS

KEY RESOURCES TABLE

REAGENT or RESOURCE	SOURCE	IDENTIFIER
Antibodies		
Rabbit anti-TH (dilution 1:200)	Millipole	Cat#AB152; RRID: AB_390204
Mouse anti-HA (dilution 1:1000)	ABclonal	Cat#AE008; RRID: AB_2770404
Goat-anti-rabbit 633 (dilution 1:1000)	Invitrogen	Cat#A-21071; RRID: AB_141419
Goat-anti-mouse (dilution 1:1000)	Invitrogen	Cat#A-21052; RRID: AB_2535719
Mouse anti-Flag (dilution 1:400)	ABclonal	Cat#AE005, RRID: AB_2770401
Rabbit anti-HA (dilution 1:10000)	Cell signaling	Cat#3724S, RRID:AB_1549585
Rabbit-HRP (dilution 1:1000).	CWbio	Cat#cw0103s, RRID:AB_2814709
Chemicals, peptides, and recombinant proteins		
Agar (w/v 1%)	Solarbio	A8190
Tryptone (w/v 1.7%)	OXOID	LP0042
Sucrose (w/v 10%)	Sinopharm Chemical Reagent	10021418
Brilliant blue	Care	N/A
Sulforhodamine B	sigma	341738
all-trans-retina (ATR)	sigma	R2500
ATP (2.5 mM)	Merck	A1852
TTX (1 μM)	TaiZhou KangTe	N/A
SCH 23390 hydrochloride (100 μM)	TOCRIS	0925
Alexa Fluor 568 hydeazide	Invitrogen	A10441
PFA	EMS	157–8
PBS	Sangon Biotech	B548117
GS	Gibco	16210–072
Triton X-100	Merck	11869
DMSO (10 mM)	sigma	D8418
Experimental models: Organisms/strains		
UAS-TH-miR#G	Xie et al.[89]	N/A
UAS-Dop1R1-miR	Xie et al.[89]	N/A
UAS-Dop1R2-miR	Xie et al.[89]	N/A
TH-C-Gal4DBD	Xie et al.[89]	N/A
DAT-B-Gal4DBD	Xie et al.[89]	N/A
R55C01-Gal4DBD	Xie et al.[89]	N/A
R60C07-Gal4DBD	Xie et al.[89]	N/A
R76F01-Gal4DBD	Xie et al.[89]	N/A
R76F05-Gal4DBD	Xie et al.[89]	N/A
TH-Gal80	Berry et al.[90]	N/A
dilp2-LexA	Li and Gong.[91]	N/A
ilp2^{1}, gd2HF	Park et al.[45]	N/A
w^{1118}	Bloomington *Drosophila* Stock Center (BDSC)	5905
R67D01-Gal4	Bloomington *Drosophila* Stock Center (BDSC)	39412
VT029577-p65AD; VT038817-Gal4DBD	Bloomington *Drosophila* Stock Center (BDSC)	86626
R55G08-Gal4	Bloomington *Drosophila* Stock Center (BDSC)	50422
R52B01-Gal4	Bloomington *Drosophila* Stock Center (BDSC)	38820
R60D05-Gal4	Bloomington *Drosophila* Stock Center (BDSC)	39247

(*Continued on next page*)

Continued		
REAGENT or RESOURCE	SOURCE	IDENTIFIER
R37F06-Gal4	Bloomington *Drosophila* Stock Center (BDSC)	49962
R55G08-LexA	Bloomington *Drosophila* Stock Center (BDSC)	53544
R52B01-LexA	Bloomington *Drosophila* Stock Center (BDSC)	52826
R60D05-LexA	Bloomington *Drosophila* Stock Center (BDSC)	52867
R37F06-LexA	Bloomington *Drosophila* Stock Center (BDSC)	52764
LexAop-Gal80	Bloomington *Drosophila* Stock Center (BDSC)	32216
dilp2-Gal4	Bloomington *Drosophila* Stock Center (BDSC)	37516
tub-Gal80^{ts}	Bloomington *Drosophila* Stock Center (BDSC)	7018
UAS-InR-CA[41]	Bloomington *Drosophila* Stock Center (BDSC)	8263
UAS-InR-DN[41]	Bloomington *Drosophila* Stock Center (BDSC)	8252
UAS-CsChrimson	Bloomington *Drosophila* Stock Center (BDSC)	55135
UAS-GtACR2	Bloomington *Drosophila* Stock Center (BDSC)	92987
LexAop-CsChrimson	Bloomington *Drosophila* Stock Center (BDSC)	55138
UAS-CaMPARI	Bloomington *Drosophila* Stock Center (BDSC)	58761
UAS-jGCaMP7s	Bloomington *Drosophila* Stock Center (BDSC)	79032
LexAop-P2X_2	Bloomington *Drosophila* Stock Center (BDSC)	76030
UAS-P2X_2	Bloomington *Drosophila* Stock Center (BDSC)	91223
LexAop-jGCaMP7s	Bloomington *Drosophila* Stock Center (BDSC)	80913
UAS-mGFP	Bloomington *Drosophila* Stock Center (BDSC)	5137
UAS-sytGFP	Bloomington *Drosophila* Stock Center (BDSC)	6925
UAS-DenMark	Bloomington *Drosophila* Stock Center (BDSC)	33061
UAS-CD4-spGFP1-10, LexAop-CD4-spGFP11	Bloomington *Drosophila* Stock Center (BDSC)	58755
UAS-nSyb-spGFP1-10, LexAop-CD4-spGFP11	Bloomington *Drosophila* Stock Center (BDSC)	64314
UAS-InR-RNAi	Vienna *Drosophila* RNAi Center (VDRC)	V992
UAS-D2R-Ri	Vienna *Drosophila* RNAi Center (VDRC)	V11471
Oligonucleotides		
Generate R67D01-p65AD: forward primer 5′- cgaaaagtgccacctgacgtcAGAA GGGGCTTTTGCAAGAAC	Integrated DNA Technologies	N/A
Generate R67D01-p65AD: reverse primer 5′- tccccgggcgagctcggccggccCCCT TGGGCCGCAATTAA	Integrated DNA Technologies	N/A
Recombinant DNA		
Plasmid: R67D01-p65AD	This paper	N/A
pBPp65ADZpUw vector	Addgene	26234
Software and algorithms		
FIJI	ImageJ (Schneider et al.)[88]	https://imagej.net/ij/download.html
GraphPad Prism 8	GraphPad Software	https://www.graphpad.com
Excel	Microsoft	https://www.microsoft.com/en-us/microsoft-365/excel

RESOURCE AVAILABILITY

Lead contact

Further information and requests for resources and reagents should be directed to and will be fulfilled by the lead contact, Yan LI (liyan@ibp.ac.cn).

Materials availability

All reagents and materials generated in this study are available from the lead contact.

Data and code availability

- All data are available from the lead author upon request.
- This paper does not report original code.
- Any additional information required to reanalyze the data reported in this paper is available from the lead contact upon request.

EXPERIMENTAL MODEL AND STUDY PARTICIPANT DETAILS

Fly strains and cultivation

Genotypes and sources of fly strains used in this paper are listed in the key resource table, and the genotypes in each figures and video are listed in Table S4. For generating R67D01-p65AD transgenic fly, the enhancer region of R67D01 was obtained from the genomic DNA of R67D01-Gal4 and cloned into the vector pBPp65ADZpUw (26234, Addgene). The resulting plasmid was injected into fly embryos and inserted into the attP40 site via phiC31 by the Core Facility of *Drosophila* Resource and Technology, Shanghai Institute of Biochemistry and Cell Biology, Chinese Academy of Sciences.

Flies were reared on normal food with the recipe of Bloomington *Drosophila* Stock Center. The proportions of different components were evaluated as approximately 1.7% protein and 10% carbohydrate. The flies were cultured at 25°C, 40–50% humidity, and 12/12 light/dark cycle, unless otherwise stated. Adult female flies were collected at hatch and aged for 3–5 days before subjected to experiments.

METHOD DETAILS

Behavior assays

Food consumption assay

All behavioral experiments were performed in a double-blinded manner. In each trial, flies of approximately 100 were collected into a bottle and aged for 3–5 days. After a starvation on agar for 24 h, these flies were subjected to a 10 min-feeding test with NF, 1.7% Tryptone, or 10% Sucrose containing 0.5% Brilliant Blue. After the test, 30 female flies from each bottle were randomly collected and divide into 3 tubes. In each tube, 10 headless flies were homogenized in 500 μL PBS and centrifuged at 12000 g for 30 min. The absorbance of the supernatant was measured at 620 nm using Multilabel Detection Platform (Hidex Chameleon Plate) and the data of the 3 tubes from the same bottle were averaged as one trial. Data from different genetic groups were normalized to the maternal control group, namely Relative Food Consumption (RFC).

In the opto-activation (CsChrimson) experiment, the flies were cultivated in the dark and fed with 0.4 mM all-trans-retina (ATR) for 1 day before experiment. The light of 623 nm (0.79 mW/mm^2) was used during test for opto-activation.

Pre-feeding assay

Pre-Feeding assay was described in our previous report.[40] Briefly, adult flies were starved for 24 h and pre-fed with agar, tryptone, or sucrose for 30 min. All groups were test for 10 min with NF containing 1% Brilliant Blue. RFC was measured and normalized to the agar groups of the maternal control group. The Suppression Index (SI) was used to quantify the feeding inhibition effect.

For PIFI, $SI_T = \frac{RFC_{Tryptone} - RFC_{Agar}}{RFC_{Agar}}$. For SIFI, $SI_S = \frac{RFC_{Sucrose} - RFC_{Agar}}{RFC_{Agar}}$.

In the opto-inhibition (GtACR2) experiment, flies were cultivated in the dark, and fed with 1mM ATP for 3 days before experiments. The light of 533 nm (30 $\mu W/mm^2$) was applied only during test period for opto-inhibition.

Two-choice assay

A group of 30 flies were tested with equal amount of two types of foods in the two-choice bottle. Flies starved in agar for 24 h were subjected to a choice bottle with 1.7% Tryptone+10% Sucrose and 10% Sucrose at two sides. Flies of protein deprivation group were fed in sucrose for 24 h and subjected to the choice between 1.7% Tryptone and 10% Sucrose. The blue dye (0.125%) and red dye (0.2%) were added to the foods alternately in parallel experiments. The tests were performed in dark, and the light of 623 nm (0.79 mW/mm^2) was applied for 30 min. The numbers of flies with blue, red or purple abdomen were counted as N_{blue}, N_{red} or N_{both}, respectively. The Choice Ratio (CR) was calculated as $CR_{blue} = \frac{N_{blue} - 0.5 \times N_{both}}{N_{total}}$ and $CR_{red} = \frac{N_{red} - 0.5 \times N_{both}}{N_{total}}$.

Climbing assay

Experimental procedure was adapted from Romero et al.[92] Ten flies were transferred into an empty vial and adapted for 10 min and gently tapped to the bottom of the vial. The number of flies climbing above 5 cm from the bottom in 10 s was counted. The experiment was repeated five times for each group under 623 nm light (0.79 mW/mm^2) or 533 nm light (30 $\mu W/mm^2$) in opto-activation or inhibition experiments, respectively.

Imaging

Immunohistochemistry and microscopy

Immunostaining experimental procedure was designed according to Wu and Luo.[93] Adult fly brains were dissected, fixed, blocked and stained with antibodies listed in the key resource table. All antibodies were diluted in blocking buffer and incubated at 4°C overnight. Confocal images were taken with Nikon A1 microscope, 20× lens and 40× oil lens using the xyz model at the resolution of 1024 × 1024 and 1 μm-step.

Syb:GRASP experiment

For syb:GRASP[52] experiments, brains were dissected, fixed and subjected to confocal imaging with identical setting. PB region was scanned under Nikon A1 microscope, 40× lens and 2× ZOOM using the xyz model at the resolution of 1024 × 512 and 1 μm-step. The PB region was selected as the ROI after the maximal intensity Z-projection using the FIJI. The mean fluorescent intensity within the PB region was subtracted by the background intensity and normalized to the average intensity of the agar group.

CaMPARI experiment

The CaMPARI experiment was performed according to Fosque et al.[44] In brief, flies were starved for 24 h and pre-fed with Agar, Tryptone, and Sucrose. Brains were immediately dissected in PBST containing 10 mM DMSO and put under the photoconversion light (405 nm, 300 mW/cm^2) for 5 min before the fixation. The cell bodies of T1-DANs were scanned under Nikon A1 microscope, 40× lens in both the green (488 nm) and red (561 nm) channels using identical imaging acquisition settings for all groups. For each neuron, the photoconversion rate was calculated by the ratio of the mean fluorescent intensity of T1-DANs in the red channel to that in the green channel.

Ex vitro *calcium imaging*

Female flies at the age of 4–7 days were starved for 16–24 h before the experiment. Single brain was dissected, transferred into the chamber, and incubated in the saline[94] with 1 μM TTX for 15 min. Immediately after the incubation, brains were subjected to calcium imaging under the Leica SP5 microscope, 20× water lens and xyt model at 1 fps. For $P2X_2$ activation, 2.5 mM ATP mixed with Alexa Fluor 568 hydeazide was delivered by the Pressure Systems for Ejection of Picoliter Volumes (Parker Picospritzer III) for 5 s. The start point of ATP application was determined by the detection of the dye. For the antagonist treatment, 100 μM SCH 23390 was incubated for 10 min before the ATP application. TTX and SCH 23390 was added to the perfusion saline once they were incubated. For each experiment, the condition of the neurons was checked by applying 85 mM KCl in saline for 5 s at the end of imaging.

ELISA

Transgenic fly *ilp2^1,gd2HF* was used to detect circulating DILP2.[45] Briefly, after pre-feeding, approximately 30 flies were quickly put into a 0.6 mL EP tube with glass fibers.[95] After a 3-min centrifuge at 9000 g in 4°C, 3 μL hemolymph collected from two parallel tubes was subjected to the ELISA test. Antibodies used in this assay were anti-Flag (ABclonal, AE005, 1:400), anti-HA (Cell signaling, C29F4, 1:10000), and Rabbit-HRP (cwbio, cw0103s, 1:1000). The 405 nm absorbance was detected by microplate readers (TECAN, Infinite F50). Hemolymph of *w^{1118}* flies was used as the blank control. The normalized circulating DILP2 levels were calculated as $\frac{A450_{Agar/Tryptone/Sucrose} - AVG\ A450_{blank}}{A450_{Agar} - AVG\ A450_{blank}}$.

QUANTIFICATION AND STATISTICAL ANALYSIS

All data were analyzed with Graphpad Prism and plotted as mean ± standard error of mean (S.E.M.). According to the data type and sample size, the data were assumed to conform normal distribution. Student's *t* test was used to compare two groups. Paired *t* test was used for the comparison between two paired measurements before and post treatment. One-way ANOVA followed by Dunnett's multiple comparisons test was applied to determine the difference among groups. two-way ANOVA was used to determine the interaction between two factors. *, $p < 0.05$; **, $p < 0.01$; ***, $p < 0.001$. All of the statistical details of experiments can be found in the figure legends.

Cell Reports, Volume *43*

Supplemental information

A brain-derived insulin signal

encodes protein satiety

for nutrient-specific feeding inhibition

Xiaoyu Li, Yang Yang, Xiaobing Bai, Xiaotong Wang, Houqi Tan, Yanbo Chen, Yan Zhu, Qili Liu, Mark N. Wu, and Yan Li

SUPPLEMENTAL INFORMATION

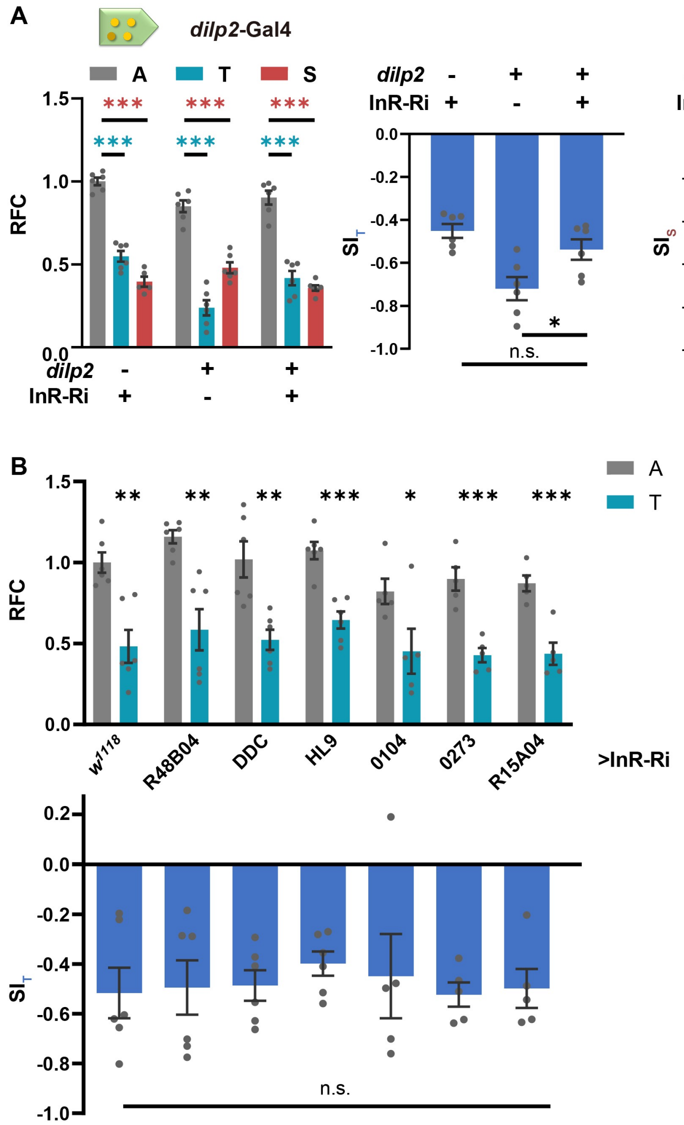

Figure S1. Insulin signalling in IPCs and some DANs is not required for feeding inhibition.

Related to Figure 1.

(A) Knockdown of InR in IPCs did not affect the PIFI or SIFI effect. n = 5-6.

(B) Knockdown of InR in various subsets of DANs did not affect the PIFI effect. n = 5-6.

n represents the number of trials. Student's *t* test for Relative Food Consumption (RFC). One-way ANOVA, Dunnett test for Suppression Index (SI). *, $p < 0.05$. **, $p < 0.01$. ***, $p < 0.001$. n.s. indicates no statistical significance. The data are shown in Mean ± S.E.M.

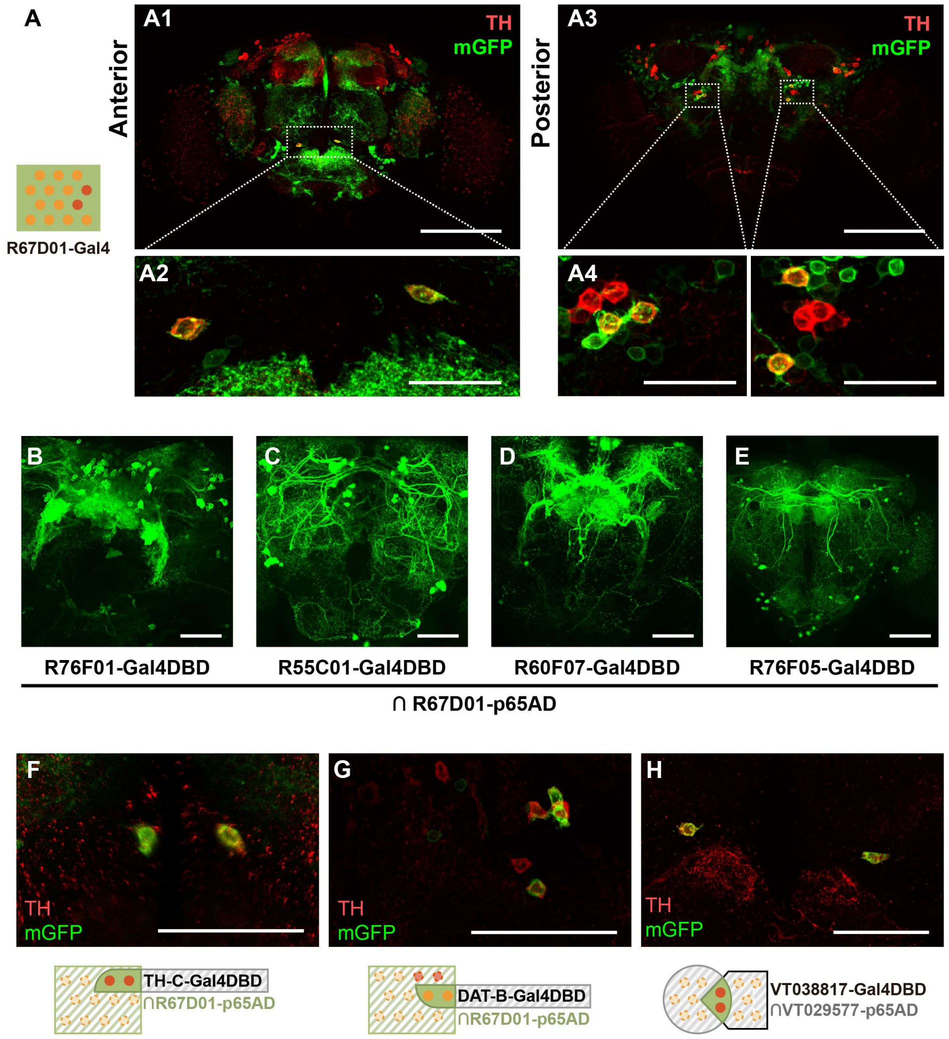

Figure S2. Identification of two types of DANs in R67D01-labeled neurons. Related to Figure 2.

(A) TH staining (red) marks the cell bodies of two types of DANs in R67D01-neurons from the anterior view (A1) and the posterior view (A3). Scale bar, 100 µm. A2 and A4 are the partial enlarged view of A1 and A3. Scale bar, 20 µm.

(B-E) The expression pattern of the split-Gal4 lines of R67D01-p65AD in combination with different Gal4DBD fly strains. Scale bar, 50 µm.

(F-H) The enlarged view of cell bodies of T1-Gal4 (Fig. 2A), PPM3-Gal4 (Fig. 2B) and T1'-Gal4 (Fig. 2C). Scale bar, 50 µm.

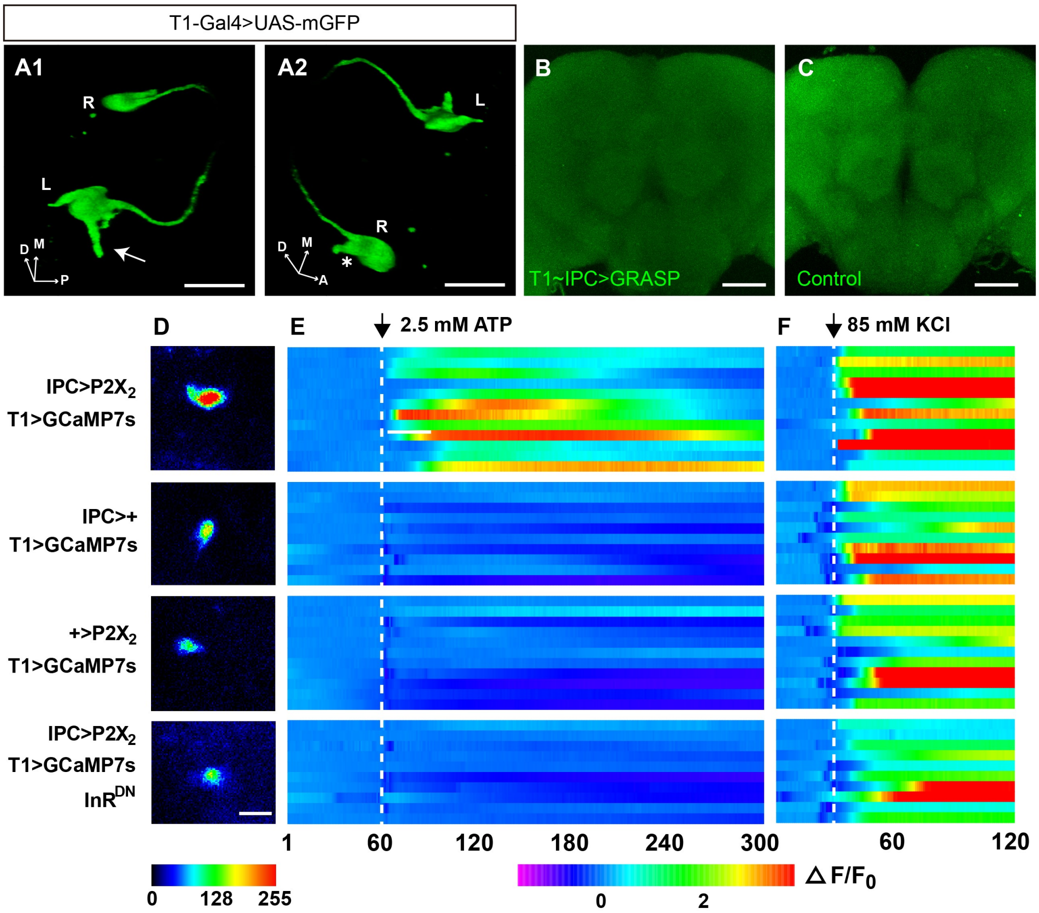

Figure S3. No synaptic but functional connection between IPCs and T1-DANs. Related to Figure 3.

(A) The 3D illustration of filipodia-like (arrow) and lamellipodia-like (star) cellular protrusion of T1-DANs. R and L indicates the right and left side, respectively. Scale bar, 10 µm.

(B-C) There was no detectable GRASP signal between IPCs and T1-DANs. Scale bar, 50 µm.

(D) Activation of IPCs induced a remarkable increase in calcium levels of T1-DANs. Representative images in different groups. Scale bar, 15 µm.

(E-F) Each row in the heat map represents the $\Delta F/F_0$ response of one T1-DANs neuron. Each pair of vertical dashed lines marks stimulation duration. The number of brains n = 10-12.

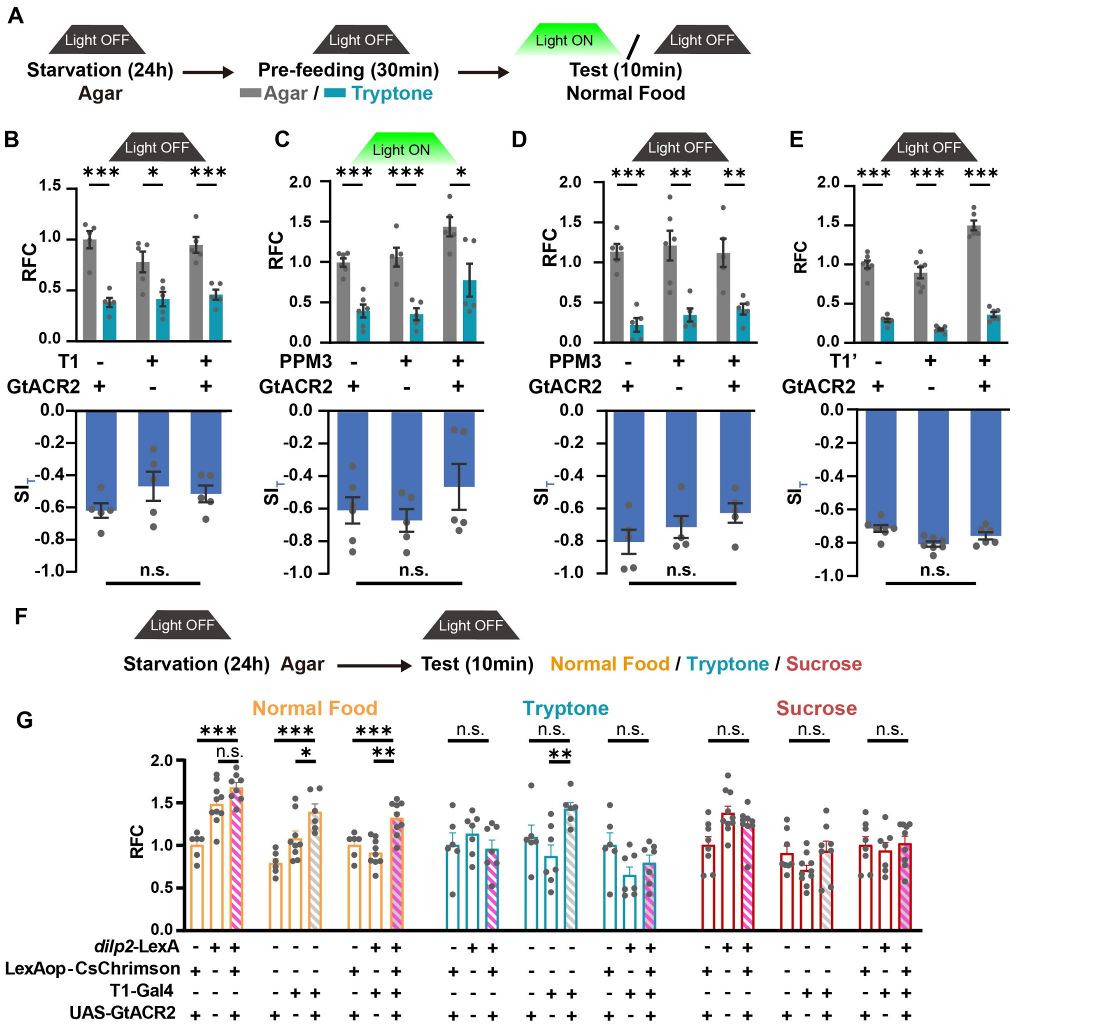

Figure S4. Silencing PPM3-DANs does not affect the PIFI effect. Related to Figure 4.

(A) The diagram of optogenetic manipulation in the pre-feeding paradigm.

(B) Flies showed comparable PIFI when light was OFF. n = 5.

(C-D) Inhibition of PPM3-DANs does not affect PIFI. n = 5-6.

(E) Flies showed comparable PIFI when light was OFF. n = 6-7.

(F-G) Flies showed comparable food consumption when light was OFF. n = 6-10. Same set of data were used for the first and seventh column in all the three groups of experiments.

n represents the number of trials. Student's *t* test for Relative Food Consumption (RFC) in B-E. One-way ANOVA, Dunnett test for Suppression Index (SI) in B-E and RFC in G. See also Table S2 for Two-way ANOVA comparison between groups. *, $p < 0.05$. **, $p < 0.01$. ***, $p < 0.001$. n.s. indicates no statistical significance. The data are shown in Mean ± S.E.M.

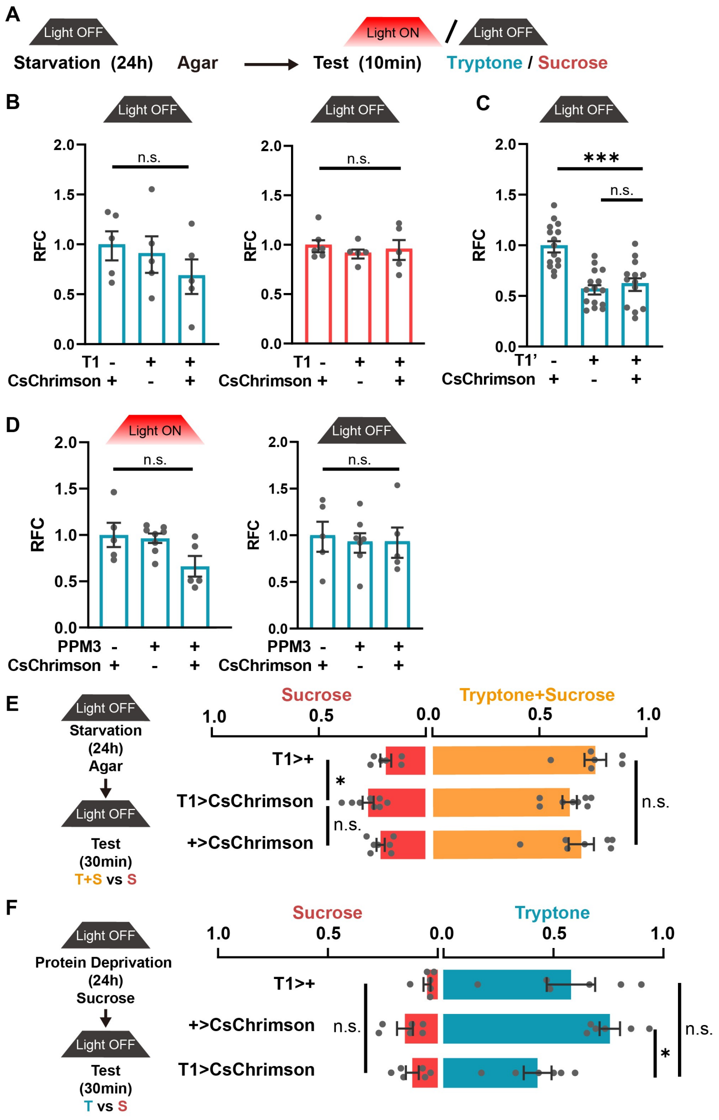

Figure S5. Activation of PPM3-DANs does not affect protein feeding. Related to Figure 5.

(A) The diagram of optogenetic manipulation in the feeding test.

(B-C) Flies showed comparable food consumption when light was OFF. n = 5-6 in B. n = 13-15 in C.

(D) Opto-activating PPM3-DANs did not affect protein intake. n = 5-8.

(E-F) Flies showed comparable choice index when light was OFF. n = 6-8.

n represents the number of trials. One-way ANOVA, Dunnett test for Relative Food Consumption (RFC) in B-D and Choice Ratio in E and F. *, $p < 0.05$. **, $p < 0.01$. ***, $p < 0.001$. n.s. indicates no statistical significance. The data are shown in Mean ± S.E.M.

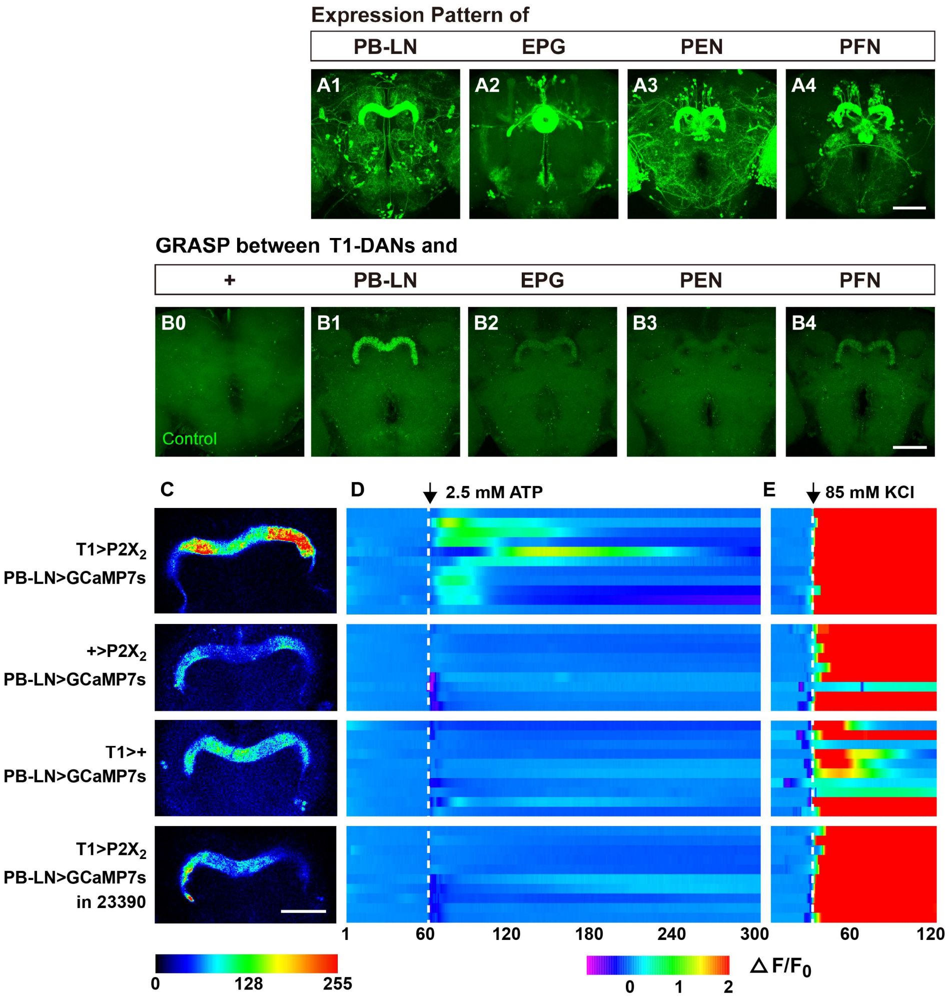

Figure S6. T1-DANs form synaptic and functional connections with PB-LNs. Related to Figure 6.

(A) The expression pattern of different types of PB-projecting neurons.

(B) The syb:GRASP between T1-DANs and PB-projecting neurons (B1-B4). B0 is the control with no PB-driver.

(C) Activation of T1-DANs induced a remarkable increase in calcium levels of PB-LNs. Representative images in different groups.

(D-E) Each row in the heat map represents the $\Delta F/F_0$ response of individual PB-LNs. Each pair of vertical dashed lines marks stimulation duration. The number of brains n = 9-11.

Scale bar, 50 μm.

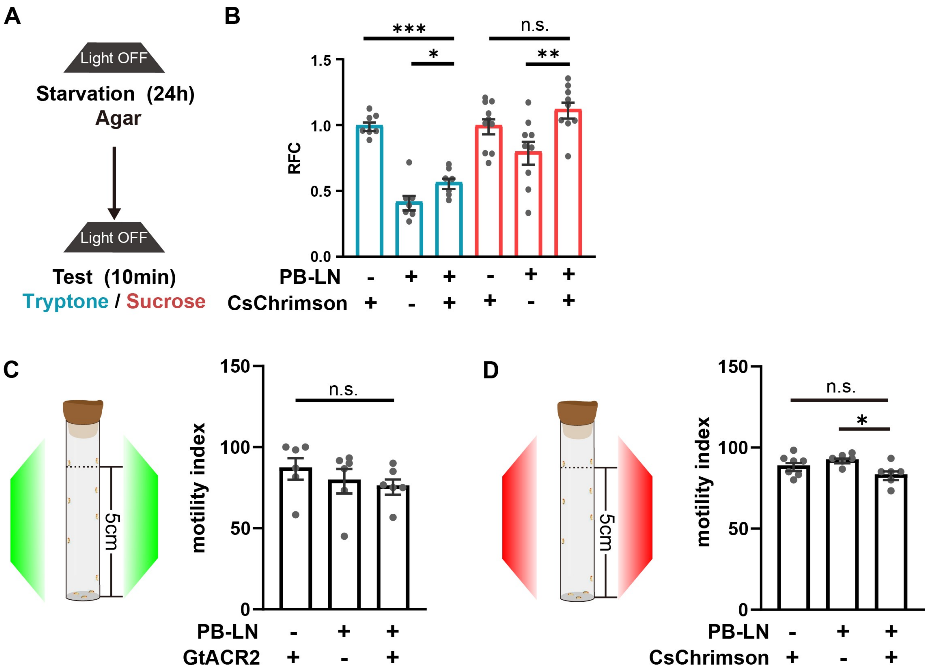

Figure S7. Manipulation of PB-LNs does not affect motility. Related to Figure 7.

(A-B) Flies showed comparable food consumption when light was OFF. n = 7-10.

(C) Silencing PB-LNs did not affect the climbing activity of flies. n = 6.

(D) Optogenetic activation of PB-LNs did not affect the climbing activity of flies. n = 6-7.

n represents the number of trials. One-way ANOVA, Dunnett test. *, $p < 0.05$. **, $p < 0.01$. ***, $p < 0.001$. n.s. indicates no statistical significance. The data are shown in Mean ± S.E.M.
